# DDR2-regulated arginase activity in ovarian cancer-associated fibroblasts promotes collagen production and tumor progression

**DOI:** 10.1038/s41388-023-02884-3

**Published:** 2023-11-23

**Authors:** Favour A. Akinjiyan, Zainab Ibitoye, Peinan Zhao, Leah P. Shriver, Gary J. Patti, Gregory D. Longmore, Katherine C. Fuh

**Affiliations:** 1 Department of Obstetrics and Gynecology, Washington University School of Medicine, St. Louis, MO 63110 USA; 2 Center for Reproductive Health Sciences, Washington University, St Louis, MO 63110 USA; 3 ICCE Institute, Washington University, St Louis, MO 63110 USA; 4 Department of Medicine (Oncology), Washington University, St. Louis, MO 63110 USA; 5 Department of Chemistry, Washington University School of Medicine, St. Louis, MO 63110 USA; 6 Center for Metabolomics and Isotope Tracing, Washington University, St. Louis, MO 63130 USA; 7 Department of Obstetrics and Gynecology & Reproductive Sciences, University of California San Francisco, San Francisco, CA 94143 USA

**Keywords:** Gynaecological cancer, Cell biology

## Abstract

Ovarian cancer has poor survival outcomes particularly for advanced stage, metastatic disease. Metastasis is promoted by interactions of stromal cells, such as cancer-associated fibroblasts (CAFs) in the tumor microenvironment (TME), with tumor cells. CAFs play a key role in tumor progression by remodeling the TME and extracellular matrix (ECM) to result in a more permissive environment for tumor progression. It has been shown that fibroblasts, in particular myofibroblasts, utilize metabolism to support ECM remodeling. However, the intricate mechanisms by which CAFs support collagen production and tumor progression are poorly understood. In this study, we show that the fibrillar collagen receptor, Discoidin Domain Receptor 2 (DDR2), promotes collagen production in human and mouse omental CAFs through arginase activity. CAFs with high DDR2 or arginase promote tumor colonization in the omentum. In addition, DDR2-depleted CAFs had decreased ornithine levels leading to decreased collagen production and polyamine levels compared to WT control CAFs. Tumor cell invasion was decreased in the presence CAF conditioned media (CM) depleted of DDR2 or arginase-1, and this invasion defect was rescued in the presence of CM from DDR2-depleted CAFs that constitutively overexpressed arginase-1. Similarly, the addition of exogenous polyamines to CM from DDR2-depleted CAFs led to increased tumor cell invasion. We detected SNAI1 protein at the promoter region of the arginase-1 gene, and DDR2-depleted CAFs had decreased levels of SNAI1 protein at the arginase-1 promoter region. Furthermore, high stromal arginase-1 expression correlated with poor survival in ovarian cancer patients. These findings highlight how DDR2 regulates collagen production by CAFs in the tumor microenvironment by controlling the transcription of arginase-1, and CAFs are a major source of arginase activity and L-arginine metabolites in ovarian cancer models.

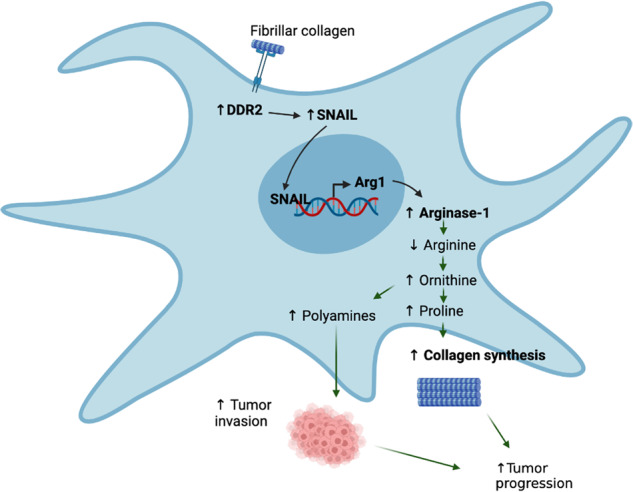

## Introduction

The process of cancer progression is supported by replacement of normal tissue matrix with tumor-associated matrix which is primarily produced by cancer-associated fibroblasts (CAFs). A major structural component of the tumor extracellular matrix (ECM) is fibrillar collagens. Their abundance, fiber orientation, and architecture have been shown to be associated with pro-tumorigenesis in multiple cancers [1–8]. This interplay between CAFs and fibrillar collagens has been shown to facilitate tumor growth and metastasis in ovarian cancer [9–11].

The receptor tyrosine kinase, Discoidin Domain Receptor 2 (DDR2), is a non-integrin collagen receptor that acts as a sensor of ECM fibrillar collagens. The action of DDR2 in CAFs can influence fibrillar collagen mRNA levels and can mechanically remodel tumor ECM collagen fibers via integrin regulation [5]. High expression of DDR2 in experimental mouse tumor models leads to increased tumor metastasis, and in various human tumors is associated with poor survival [12–17].

L-arginine is a nonessential amino acid that is cleaved by arginase to urea and L-ornithine. L-ornithine can then be further metabolized by ornithine decarboxylase (ODC) to polyamines or by ornithine aminotransferase (OAT) to form proline. Proline is a critical and abundant amino acid in the biosynthesis of collagens. Arginase has largely been studied in the immune response in myeloid cells as well as modulating T cell immunity [18–20]. In tumors, arginase is highly expressed in tumor-associated macrophages (TAMs) and myeloid derived suppressor cells (MDSCs) which deplete available arginine leading to T cell impairment [21]. However, the regulation and functional role of arginase expression by CAFs has not been extensively evaluated. Here we report that DDR2 regulates collagen protein production by CAFs in the tumor microenvironment by controlling the transcription of arginase-1. Our findings reveal how CAFs are a major source of arginase activity and L-arginine metabolites in ovarian tumors and that DDR2 and arginase in CAFs may be a target in ovarian cancer.

## Results

### DDR2-null mice have decreased ovarian tumor burden

**Fig. 1:**
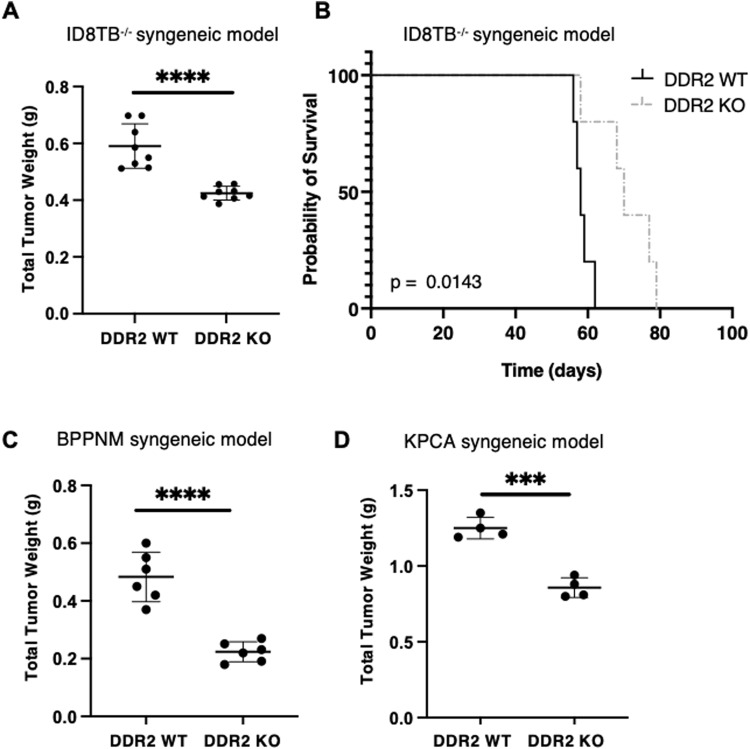
Deletion of host DDR2 leads to decrease in tumor burden. **A** DDR2-positive ID8 *Trp53*^−/−^ *Brca2*^−/−^ (ID8TB^−/−^) mouse ovarian surface epithelium cell (MOSEC) tumor line was intraperitoneally injected into C57BL/6J *Ddr*2 WT mice (8 mice; *n* = 8) or *Ddr*2 KO mice (8 mice; *n* = 8). **B** Kaplan–Meier survival curves for C57BL/6J *Ddr*2 WT (5 mice; *n* = 5) or *Ddr*2 KO (5 mice; *n* = 5) mice injected intraperitoneally with ID8TB^−/−^ tumor line and monitored till disease endpoint. **C** DDR2-positive BPPNM (*p53*^−/−R172H^*Brca1*^−/−^Pten^−/−^Nf1^−/−^Myc^OE^) mouse fallopian tube epithelial (FTE) tumor cell line was intraperitoneally injected into C57BL/6J *Ddr*2 WT mice (6 mice; *n* = 6) or *Ddr*2 KO mice (6 mice; *n* = 6). (D) DDR2-negative KPCA (p53^−/−R172H^Ccne1^OE^Akt2^OE^KRAS^G12V^) mouse FTE tumor cell line was intraperitoneally injected into C57BL/6J *Ddr*2 WT mice (4 mice; *n* = 4) or *Ddr*2 KO mice (4 mice; *n* = 4). In panels **A**, **C**, and **D**, Student’s *t*-test was used for statistics ****p < 0.0001, ***p < 0.001. In panel **B**, Logrank (Mantel Cox) test was used for analysis.

Immunohistochemical evaluation of advanced stage, human ovarian cancer specimens demonstrate that high expression of DDR2 in tumors is associated with poor survival [22]. Since DDR2 is predominantly expressed by mesenchymal cells, we asked whether presence of DDR2 in ovarian cancer-associated stromal cells impacted ovarian tumor burden in mouse models. To do so, we utilized a previously published intraperitoneal tumor model by introducing three mouse ovarian tumor cell lines into the intraperitoneal cavity of adult female Ddr2^−/−^ or control WT C57BL/6 mice (i.e., a syngeneic mouse tumor model). The three ovarian cancer cell lines were: (1) ID8TB^−/−^ (mouse ovarian surface epithelium cell line [23]), (2) BPPNM (fallopian tube epithelial-derived cell line [24]), and (3) KPCA (fallopian tube epithelial-derived cell line [24]). ID8TB^−/−^ and BPPNM tumor cell lines expressed DDR2, while KPCA did not (Supplementary Fig. S1A). In all three experimental settings, ubiquitous Ddr2^−/−^ recipient mice developed significantly less tumor burden than the WT mice, and the ID8TB^−/−^ model in DDR2 KO mice had increased survival and decreased ascites compared to the DDR2 WT mice (Fig. 1A–D, Supplementary Fig. S1B). These data indicated that the presence of DDR2 in stromal cells or the host, in general, impacted ovarian cancer burden, regardless of tumor cell DDR2 expression status.

### Arginase-1 mRNA expression is decreased in tumors from Ddr2^−/^^−^ mice

**Fig. 2:**
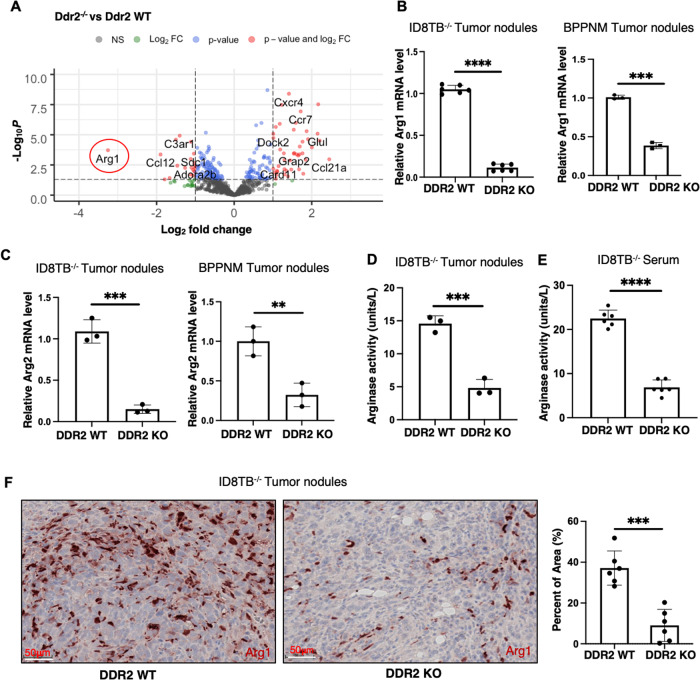
Arginase-1 is downregulated in tumor nodules from DDR2 KO mice. **A** Volcano plot showing differentially expresses genes in RNA from ID8TB^−/−^ tumor nodules from Ddr2 WT mice (5 mice; *n* = 5) or Ddr2^−/−^ mice (5 mice; *n* = 5). **B** Quantitative PCR assay showing Arg1 gene expression using RNA from ID8TB^−/−^ (6 mice; *n* = 6) and BPPNM (3 mice; *n* = 3) tumor nodules from Ddr2 WT mice or Ddr2^−/−^ mice. **C** Quantitative PCR assay showing Arg2 gene expression using RNA from ID8TB^−/−^ and BPPNM tumor nodules from Ddr2 WT mice (3 mice; *n* = 3) or Ddr2^−/−^ mice (3 mice; *n* = 3). **D** Arginase activity assay on tumor nodules from ID8TB^−/−^ tumor-bearing Ddr2 WT mice (3 mice; *n* = 3) or Ddr2^−/−^ mice (3 mice; *n* = 3). **E** Arginase activity assay on serum from ID8TB^−/−^ tumor-bearing Ddr2 WT mice (6 mice; *n* = 6) or Ddr2^−/−^ mice (6 mice; *n* = 6). **F** Immunohistochemistry images for Arginase-1 expression on ID8TB^−/−^ tumor nodules from Ddr2 WT mice (6 mice; *n* = 6) or Ddr2^−/−^ mice (6 mice; *n* = 6). Scale bars = 50 μm (left). One tumor slice per mouse (total of 6 tumor slices per group) was used and the entire tumor slice was analyzed using Halo. Graph on right shows percent of area positive for Arg1. In all panels, Student’s *t*-test was used for statistics ****p < 0.0001, ***p < 0.001, **p < 0.01.

To determine how tumor-associated stromal expression of DDR2 affected tumor burden, we performed targeted mRNA expression profiling of ID8TB^−/−^ tumors dissected from WT and Ddr2^−/−^ mice using the Nanostring nCounter Tumor Signaling 360 panel which includes 760 genes and 20 internal reference genes. Volcano plot analysis revealed that arginase-1 (Arg1) mRNA level, in particular, was dramatically decreased in tumors from Ddr2^−/−^ hosts (Fig. 2A, Supplementary Fig. S2A, Supplementary Table 2). Quantitative PCR on mRNA isolated from omental ID8TB^−/−^ tumor nodules, different than those used for Nanostring analysis, confirmed that Arg1 mRNA was indeed decreased in ID8TB^−/−^ and BPPNM tumor nodules from Ddr2^−/−^ hosts (Fig. 2B). Related Arg2 mRNA level was also decreased in tumor nodules (Fig. 2C). Arginase enzyme activity in whole tumor extracts and serum from tumor-bearing mice was also significantly decreased in Ddr2^−/−^ mice (Fig. 2D, E). Finally, when fixed tumor slices were immunostained for arginase-1 protein, tumors from Ddr2^−/−^ hosts had decreased arginase-1 expression (Fig. 2F).

### The action of DDR2 in CAFs controls arginase activity

**Fig. 3:**
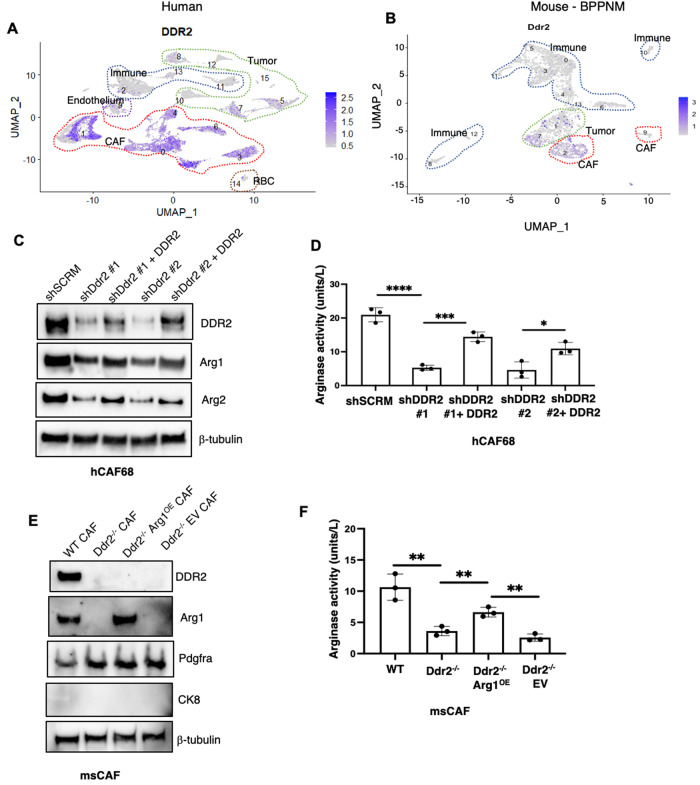
DDR2 regulates arginase activity in omental cancer-associated fibroblasts (CAFs). **A** UMAP plot showing DDR2 expression in various cell type clusters from human ovarian cancer samples from PMID: 34238352. **B** UMAP plot showing Ddr2 expression in various cell type clusters from BPPNM mouse tumors from PMID: 33158843. **C** Western blot of DDR2-expressing (shSCRM), DDR2-depleted (shDdr2) and DDR2 rescue (shDdr2 + DDR2 rescue) CAFs with the indicated antibodies. Two separate short-hairpin RNA targeting DDR2 (#1 and #2) were used. **D** Arginase activity assay of DDR2-expressing (shSCRM), DDR2-depleted (shDdr2) and DDR2 rescue (shDdr2 + DDR2 rescue) CAFs (*n* = 3). **E** Western blot of CAFs from ID8 Trp53^−/−^ Brca2^−/−^ tumor-bearing DDR2 WT and Ddr2^−/−^ mice as well as DDR2-null CAFs with constitutive overexpression of arginase-1 (Ddr2^−/−^Arg1^OE^) or control vector (Ddr2^−/−^EV) with the indicated antibodies. **F** Arginase activity assay on CAFs from ID8 Trp53^−/−^ Brca2^−/−^ tumors from DDR2-expressing (WT) and DDR2-null (Ddr2^−/−^) mice as well as DDR2-null CAFs with constitutive overexpression of arginase-1 (Ddr2^−/−^Arg1^OE^) or control vector (Ddr2^−/−^EV) (*n* = 3). In all panels, Student’s *t*-test was used for statistics ****p < 0.0001, ***p < 0.001, **p < 0.01, *p < 0.05.

To determine which cell(s) in the host tumor stromal compartment expressed DDR2, we first interrogated published human and BPPNM mouse ovarian cancer single-cell RNA sequencing datasets [24]. In both, DDR2 was found to be primarily expressed in CAFs but was also present in some tumor cell clusters (Fig. 3A, B, Supplementary Fig. S3A and B, Supplementary Tables 3 and 4). Notably, in both samples, none of the identified immune cell clusters expressed DDR2 mRNA.

Based on these results, we examined three distinct validated human omental CAF cell lines for DDR2 expression [25], and all expressed DDR2 (Fig. 3C and Supplementary Fig. S3C). When DDR2 expression was shRNA-depleted in all CAF cell lines, to varying degrees (Fig. 3C and Supplementary Fig. S3C), Arg1 mRNA expression decreased (Supplementary Fig. S3D) as did cellular arginase activity (Fig. 3D and Supplementary Fig. S3E). Importantly, these changes in Arg1 expression and activity were rescued, to levels approximating that in WT CAFs by expressing a RNAi-resistant isoform of DDR2 in hCAF68 cells depleted of Ddr2 (Fig. 3C, D).

To determine if DDR2 regulates Arg1 expression and arginase activity in omental CAFs in vivo, we made use of CAFs from the mouse ID8TB^−/−^ syngeneic ovarian tumor model. CAFs from WT and Ddr2^−/−^ tumor-bearing mice were isolated as previously published [26]. Following negative selection to deplete immune cells with an anti-CD45 antibody and epithelial cells with an anti-EpCAM antibody, remaining stromal cells were immortalized using SV40 large T virus. Similar to human omental CAF cell lines, mouse CAFs from ID8TB^−/−^ tumors in Ddr2^−/−^ mice had decreased Arg1 mRNA levels (Supplementary Fig. S3F), Arg1 expression, and arginase activity (Fig. 3E, F) which was increased upon constitutive Arg1 overexpression in Ddr2^−/−^ CAFs (Fig. 3E, F).

**Fig. 4:**
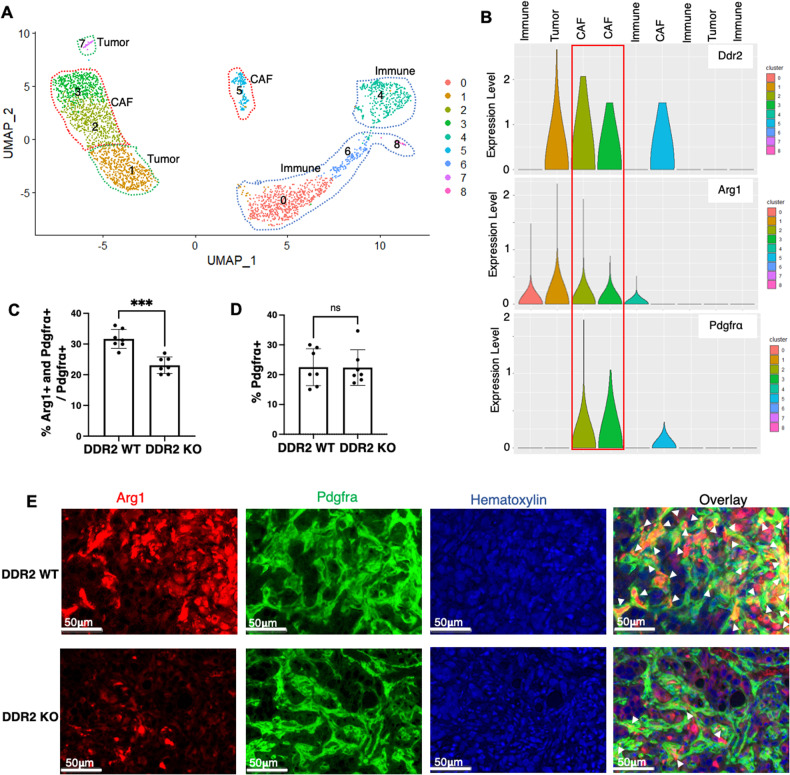
Identification of a subpopulation of DDR2-expressing CAFs that express arginase-1 in vivo. **A** UMAP plot showing showing cell clusters for ID8TB^−/−^ tumors in WT mice. **B** Violin plots showing expression of Ddr2, Arg1 and Pdgfra in cell clusters for ID8TB^−/−^ tumors in WT mice. **C** Analysis of multiplex immunohistochemistry on ID8TB^−/−^ tumor slices showing the percent of double positive Arg1+ and Pdgfra+ cells as a proportion of all Pdgfra+ cells in tumor slices (*n* = 7 mice). Entire tumor slice was analyzed in Halo. **D** Analysis of multiplex immunohistochemistry on ID8TB^−/−^ tumor slices showing the percent of Pdgfra+ cells in tumor slices (*n* = 7 mice). Entire tumor slice was analyzed in Halo. **E** Representative images of multiplex immunohistochemistry ID8TB^−/−^ tumor slices from WT and Ddr2^−/−^ mice stained for Pdgfra, Arg1 and hematoxylin (*n* = 7 mice). Scale bar = 50 μm. In all panels, Student’s *t*-test was used for statistics ***p < 0.001, **p < 0.01, ns = *p* > 0.05.

Given the findings that Arg1 is expressed in DDR2+ CAFs, we used another in vivo approach to identify whether other cell populations expressed Arg1. We performed single-cell mRNA sequencing (scRNAseq) on ID8TB^−/−^ mouse tumors dissected from WT mice (Fig. 4A, Supplementary Table 5). Using established tumor, CAF, and immune markers [24] (Supplementary Fig. S4A), we identified two tumor, three CAF and four immune cell clusters (Fig. 4A). Violin plot analysis of the various cell clusters present revealed that DDR2 was expressed in the three PDGFRΑ+ CAF clusters and one tumor cell subpopulation (Fig. 4A, B). Arg1 mRNA expression was present in two of the three Ddr2+ CAF clusters as well as in two immune cell clusters and one tumor cell cluster (Fig. 4B).

Next, we performed multiplex immunohistochemistry analysis on ID8TB^−/−^ tumors from WT and Ddr2^−/−^ mice for expression of Arg1 and various tumor stromal cell type markers (CAF – PDGFRα; macrophage – F4/80). Arg1 expression was present in 30% of cells expressing the CAF marker protein PDGFRα (Fig. 4C–E). In tumors from Ddr2^−/−^ mice, the proportion of Arg1-positive CAFs (%Arg1+ and PDGFRα+/ PDGFRα+) was significantly decreased compared to WT mice (Fig. 4C). This was not a result of overall decreased CAF populations in tumors from Ddr2^−/−^ mice as the proportion of PDGFRα+ CAFs were similar between tumors from WT and Ddr2^−/−^ mice (Fig. 4D). The proportion of Arg1-positive macrophages (%Arg1+ and F4-80+/ F4-80+) and Arg1-positive tumor cells (%Arg1 and CK8+/CK8+) were similar between tumors from WT and Ddr2^−/−^ mice (Supplementary Fig. S4B–G). Flow analysis of single-cell suspensions from ID8TB^−/−^ tumors in WT hosts revealed that 17.4% of total cells were CAFs (e.g., PDGFRα positive) (Supplementary Fig. S4H, Supplementary Table 6) while 20% of cells isolated from tumors were CD45+ immune cells (Supplementary Fig. S4H).

Taken together, these accumulated cell line and in vivo data indicate that Arg1 is expressed in Ddr2+ CAFs, and DDR2 regulates arginase-1 protein levels and arginase activity. Moreover, in this mouse ovarian tumor model, CAF-derived Arg1, as opposed to immune cell- or tumor cell-derived Arg1, was likely a significant contributor to overall arginase activity in ovarian cancer.

### Ovarian tumor omental CAFs with high DDR2 and ARG1 expression promote in vivo omental colonization

**Fig. 5:**
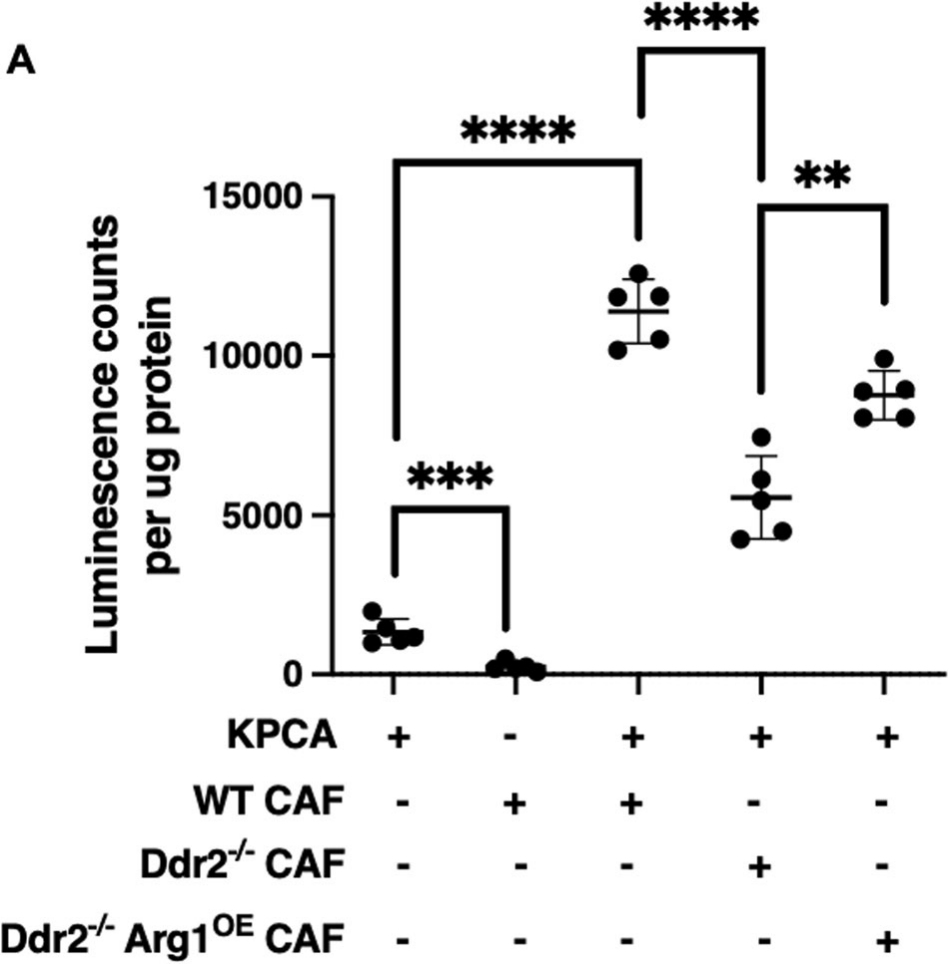
Omental CAFs with high DDR2 and/or arginase-1 expression promote in vivo omental colonization. **A** Normalized luminescence counts of omental tissue from KPCA tumor cells alone (lane 1), ID8TB^−/−^ WT mouse CAFs alone (lane 2), WT mice injected with KPCA and WT mouse CAFs (lane 3), KPCA and Ddr2^−/−^ mouse CAFs (lane 4), and KPCA and Ddr2^−/−^ mouse CAFs with Arg1 constitutive overexpression (lane 5) (*n* = 5 mice). In this experiment, luciferase-positive KPCA tumor cells were used and CAFs are negative for luciferase. Student’s *t*-test was used for statistics ****p < 0.0001, ***p < 0.001, **p < 0.01.

Omental colonization can be part of tumor progression in ovarian cancer. To determine if DDR2-regulated arginase-1 activity in CAFs impacted tumor cell colonization in vivo, we co-injected WT mice intraperitoneally with syngeneic luciferase-positive KPCA tumor cells (low DDR2 expression and low arginase activity) (Supplementary Fig. S1A, Supplementary Fig. S5A) +/− various luciferase-negative mouse omental CAF cell lines from WT or Ddr2^−/−^ mice. After 5 days, mice were sacrificed, omentum digested, and luciferase assay performed which reflected the amount of KPCA tumor cells that had colonized the omentum (Supplementary Fig. S5B). When KPCA cells were co-injected with WT mouse CAFs, there was a significant increase in omental colonization by KPCA cells (Fig. 5). Compared to WT CAFs, when Ddr2^−/−^ CAFs were used, there was significantly less tumor cell colonization (Fig. 5). Mice co-injected with Ddr2^−/−^ Arg1^OE^ CAFs had increased omental colonization compared to those co-injected with Ddr2^−/−^ CAFs. This data suggested that in vivo DDR2 and Arg1 expressing CAFs might impact early steps of omental colonization or the proliferation of tumor cells after attachment.

### SNAIL protein was detected at the promoter region of arginase-1 gene

**Fig. 6:**
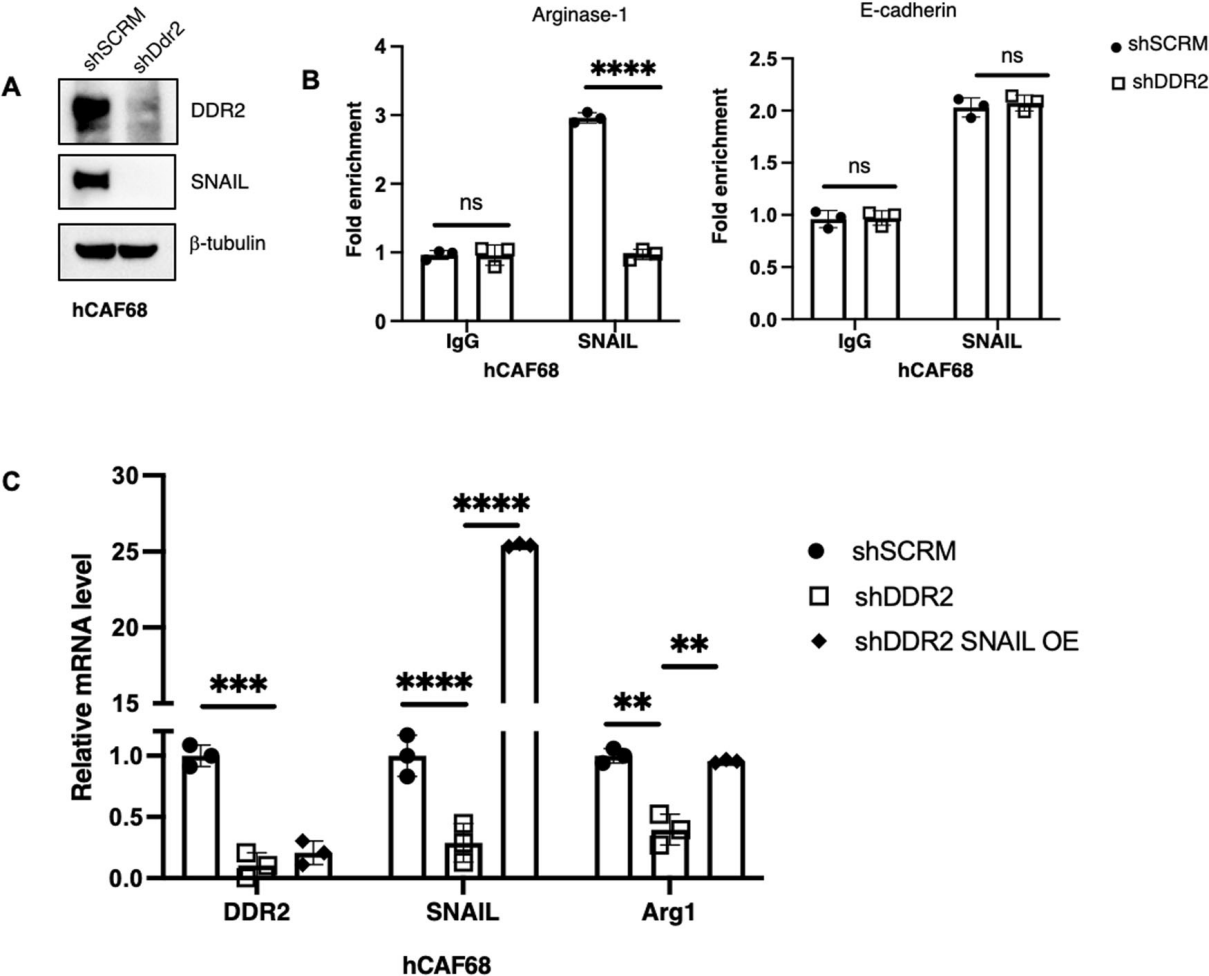
DDR2’s regulation of arginase-1 is dependent on SNAI1 transcriptional activity. **A** Western blot of DDR2-expressing (shSCRM) and DDR2-depleted (shDdr2) CAFs with the indicated antibodies. **B** Chromatin immunoprecipitation-qPCR (ChIP-qPCR) of Arg1 expression using SNAIL or IgG control antibodies on shSCRM and shDdr2 CAFs. E-cadherin expression was used as a positive control. ChIP-qPCR of E-cadherin expression using SNAIL or IgG control antibodies on shSCRM and shDdr2 CAFs (*n* = 3 replicates). **C** Quantitative PCR assay showing DDR2, SNAIL and Arg1 gene expression using RNA from shSCRM, shDDR2 and DDR2-depleted SNAIL-overexpressing CAFs (shDDR2 SNAIL OE). In all panels, ****p < 0.0001, ***p < 0.001, **p < 0.01, ns = *p* > 0.05.

The action of DDR2 in CAFs appeared to regulate Arg1 expression at the transcriptional level (Fig. 2A, B). We have previously shown that SNAIL (SNAI1), an EMT inducing transcription factor that promotes tumor cell migration and invasion [27], is regulated by the action of DDR2 in tumors, post-transcriptionally [28]. SNAI1 can act as both a transcriptional repressor and activator [29–31]. We confirmed that SNAIL protein level was indeed decreased in Ddr2-depleted CAFs (Fig. 6A). To determine if SNAIL protein could impact Arg1 transcription, we performed chromatin immunoprecipitation (ChIP) experiments to determine if SNAIL was present at the promoter region of the endogenous Arg1 gene. In human ovarian tumor CAFs, SNAIL protein was detected at the promoter region of the human Arg1 gene, while in Ddr2-depleted CAFs, there was less SNAIL detected (Fig. 6B, Supplementary Fig. S6A). To confirm this finding, we constitutively overexpressed SNAIL1 in DDR2-depleted CAFs (shDDR2 SNAIL OE) (Fig. 6C) and performed quantitative PCR for Arg1. Arg1 mRNA levels were increased in shDDR2 SNAIL OE CAFs compared to DDR2-depleted CAFs (shDDR2). In control experiments, we confirmed nuclear localization of SNAIL in shDDR2 SNAIL OE CAFs (Supplementary Fig. S6B) This suggested that DDR2-regulated SNAIL1 expression impacts Arg1 transcription in ovarian CAFs.

### DDR2-dependent arginase activity in CAFs is important for ovarian tumor collagen protein production and secretion

**Fig. 7:**
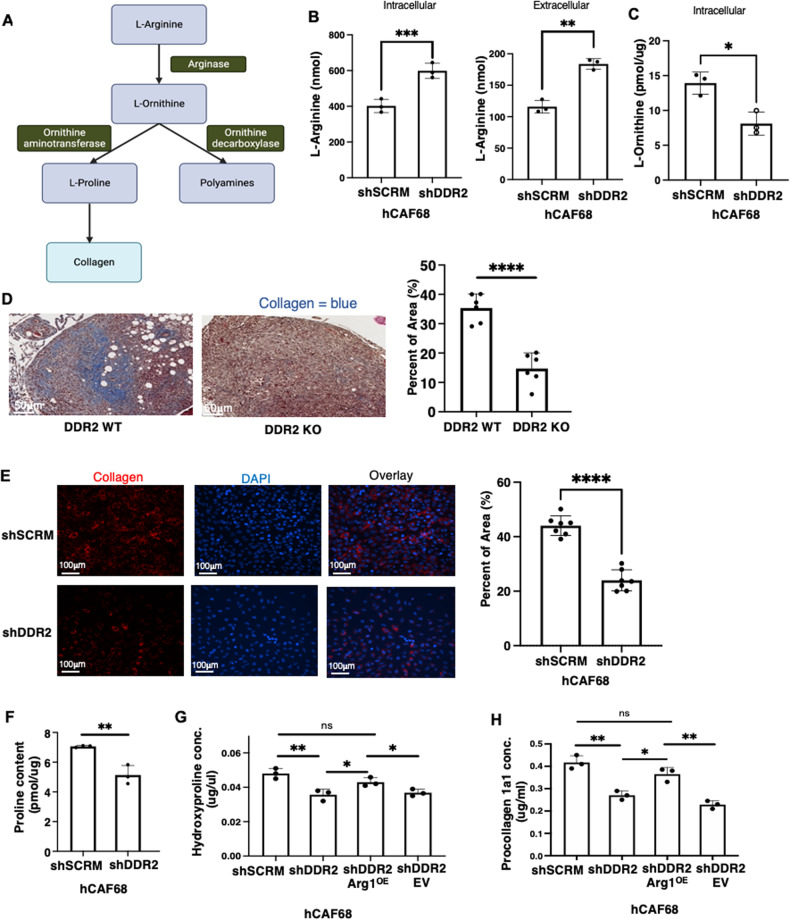
DDR2-dependent arginase activity in CAFs promotes increased collagen synthesis. **A** Schematic showing arginine metabolism by arginase into L-ornithine and other downstream products. **B** Arginine assay showing levels of intracellular and extracellular arginine in DDR2-expressing (shSCRM) and DDR2-depleted (shDdr2) CAFs (*n* = 3). **C** Ornithine assay showing levels of intracellular ornithine in DDR2-expressing (shSCRM) and DDR2-depleted (shDdr2) CAFs (*n* = 3). **D** Representative images of modified Masson’s trichrome stain for collagen (blue) from ID8 Trp53^−/−^ Brca2^−/−^ tumors from Ddr2 WT and Ddr2 KO mice (*n* = 6 mice). Scale bar = 50 μm. One tumor slice per mouse was used (6 total tumor slices per group). Percent area of collagen was quantified using the entire tumor slice in Halo. **E** Representative images of collagen immunofluorescence stain in DDR2-expressing (shSCRM) and DDR2-depleted (shDdr2) CAFs (*n* = 3 replicates, 12 images per group). **F** Proline assay showing levels of intracellular proline in DDR2-expressing (shSCRM) and DDR2-depleted (shDdr2) CAFs (*n* = 3). **G** Hydroxyproline assay measurement of DDR2-expressing (shSCRM), DDR2-depleted (shDdr2), DDR2-depeleted and constitutive arginase-1 overexpressing (shDdr2 Arg1^OE^) and DDR2-depleted empty vector control (shDdr2 EV) (*n* = 3 replicates). **H** Procollagen1a1 assay measurement of conditioned media from shSCRM, shDdr2, shDdr2 Arg1^OE^ and shDdr2 EV CAFs (*n* = 3 replicates). In all panels, Student’s *t*-test was used for statistics ****p < 0.0001, ***p < 0.001, **p < 0.01, *p < 0.05, ns = *p* > 0.05.

Arg1 is a central cytosolic enzyme controlling cellular L-Arginine metabolism. Arg1 cleaves L-Arg to generate urea and L-Ornithine. L-Ornithine is subsequently metabolized to generate L-Proline and polyamines [32–34] (Fig. 7A). To determine if DDR2 signaling impacted L-Arginine metabolite production in ovarian tumor CAFs, we generated a series of human omental CAF cells: (1) DDR2-expressing WT control (shSCRM), (2) DDR2-depleted (shDdr2), (3) DDR2-depleted and constitutively overexpressing Arg1 (shDdr2 Arg1^OE^) and (4) a transfection control empty vector (shDdr2 EV) (Supplementary Fig. S7A, B). In Ddr2-depleted CAFs, both intracellular and secreted L-Arginine levels, as determined by a standard biochemical assay, were increased (Fig. 7B). Compared to WT CAFs, L-Ornithine levels were significantly decreased in Ddr2-depleted CAFs (Fig. 7C).

In breast tumor CAFs, the action of DDR2 has been shown to contribute to the production of collagens, by affecting mRNA synthesis [13]. However, whether DDR2 signals could also regulate the production of collagen proteins, and if so, how has not been addressed. When ovarian tumor nodules from WT and Ddr2^−/−^ mice were stained for fibrillar collagens with trichrome blue, the amount of detected fibrillar collagen in tumors from Ddr2^−/−^ mice was significantly decreased (Fig. 7D). In addition, cultured DDR2-depleted human omental CAFs expressed decreased collagen1α1 protein as detected by immunofluorescence (Fig. 7E). We next determined the L-Proline content in human omental CAF cell lines using a standard biochemical assay. Ddr2-depleted CAFs had decreased L-Proline content (Fig. 7F). In collagen proteins, much of the proline exists in its hydroxylated form, hydroxyproline [35]. In Ddr2-depleted CAFs, there was also decreased cellular hydroxyproline (Fig. 7G). Constitutive Arg1 overexpression in Ddr2-depleted cells rescued hydroxyproline levels to that present in control WT CAFs (Fig. 7G).

While hydroxyproline is a sensitive marker for collagen level in cells, it is not a direct measure of collagen synthesis since hydroxyproline residues may be elevated due to collagen synthesis and degradation. Newly synthesized triple helical procollagen is secreted into the extracellular space where additional cleavage and crosslinking occurs to form mature collagen fibers [36]. To determine if DDR2 signals affected collagen protein synthesis and secretion, we measured secreted procollagen 1α1 levels in the culture media from various CAFs. Ddr2-depleted CAFs secreted less procollagen 1α1 compared to WT CAFs (Fig. 7H). Constitutive Arg1 overexpression in Ddr2-depleted CAFs (shDDR2 Arg1^OE^) increased the amount of procollagen 1α1 secreted to levels produced by control WT CAFs (Fig. 7H). In other control experiments, siRNA-mediated depletion of Arg1 in WT CAFs resulted in decreased procollagen 1α1 secretion (Supplementary Fig. S7C, D).

To confirm that L-arginine was able to be converted to collagen in both WT and Ddr2-depleted CAFs, we performed arginine metabolic tracing experiments. Human omental CAFs (+/− DDR2) were loaded with ^13^C C6-labeled L-arginine and after 72 h intracellular collagen peptides were isolated following cell lysis and subjected to mass spectrometry. This type of experiment does not distinguish the levels of labeled collagen between different cells. As expected based on prior literature [37] in both WT and Ddr2-depleted CAFs we identified 21 collagen peptides from 11 distinct collagens that contained ^13^C C5-labeled proline or hydroxyproline (Supplementary Table 7) in both DDR2-depleted and WT control.

To determine if fibrillar collagen increases DDR2 expression in CAFs, we cultured DDR2-expressing and DDR2-depleted CAFs on plastic or polymerized collagen and checked DDR2 expression after 24 h. We observed a modest increase in DDR2 protein levels in CAFs cultured on polymerized collagen compared to CAFs cultured on plastic (Supplementary Fig. S7E).

In sum, these accumulated data indicated that DDR2-dependent regulation of arginase activity in omental CAFs contribute to collagen protein synthesis and secretion. This could explain, in part, why Ddr2^−/−^ ovarian cancer tumor nodules contain less ECM fibrillar collagens, and possibly as a result, have decreased tumor burden in vivo. Additionally, collagen may increase DDR2 levels in CAFs, suggesting the possibility of a feedback loop between collagen and DDR2.

### DDR2-dependent arginase polyamine production in CAFs contributes to tumor cell invasion

**Fig. 8:**
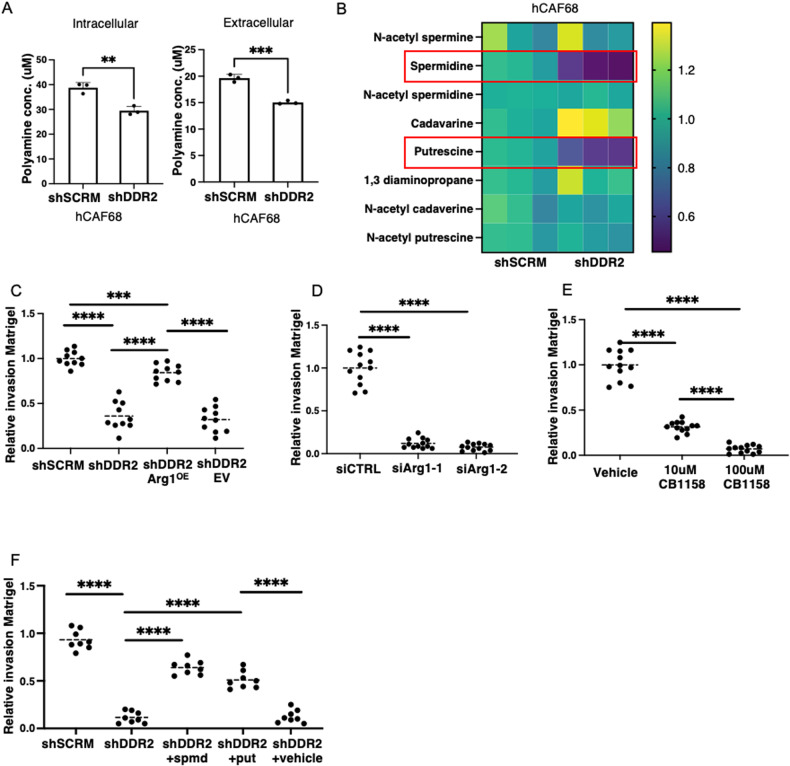
Polyamine-mediated and DDR2-dependent arginase activity in CAFs promotes tumor invasion. **A** Total polyamine assay showing levels of intracellular and extracellular polyamines in DDR2-expressing (shSCRM) and DDR2-depleted (shDdr2) CAFs and CAF conditioned media, respectively (*n* = 3 replicates). **B** Heatmap showing fold change expression of each polyamine and intermediate from mass spectrometry intensity data (*n* = 3 replicates). **C** Matrigel invasion assay of Tyknu tumor cells using conditioned media from WT control (shSCRM), DDR2-depleted (shDdr2), DDR2-depeleted and constitutive arginase-1 overexpressing (shDdr2 Arg1^OE^) and DDR2-depleted empty vector control (shDdr2 EV) as chemoattractant (*n* = 10 images analyzed from 3 replicates per condition). **D** Matrigel invasion assay of Tyknu tumor cells using conditioned media from siCTRL, siArg1-1 and siArg1-2 CAFs (*n* = 12 images analyzed from 3 replicates per condition). **E** Matrigel invasion assay of Tyknu tumor cells using conditioned media from CAFs treated with PBS solvent control (Vehicle), 10 μM or 100 μM CB1158 (*n* = 12 images analyzed from 3 replicates per condition). **F** Matrigel invasion assay of Tyknu tumor cells using CAF conditioned media from WT control (shSCRM), DDR2-depleted (shDdr2), DDR2-depleted CM supplemented with 10 μM spermidine (shDdr2 + spmd), DDR2-depleted CM supplemented with 10 μM putrescine (shDdr2 + put) and solvent control (shDdr2 + PBS). (*n* = 12 images analyzed from 3 replicates per condition). In all panels, Student’s *t*-test was used for statistics ****p < 0.0001, ***p < 0.001, **p < 0.01.

Ovarian tumor progression is dependent upon tumor cell attachment to and invasion through the basement membrane with subsequent interaction with CAFs. Given our findings that DDR2 inactivated CAFs have lower L-Ornithine levels and this can lead to a decrease in polyamines, we asked whether DDR2-regulated arginase activity in CAFs impacted polyamine production. To do this, we biochemically determined the total polyamine level in CAFs cells (+/− DDR2) and their secreted media. Ddr2-depleted CAFs had decreased intracellular and extracellular polyamine levels compared to WT control (Fig. 8A). We next performed mass spectrometry on conditioned media produced by human CAFs (+/− DDR2). Ddr2-depleted (shDDR2) CAFs produced decreased levels of spermidine and putrescine compared to WT control (shSCRM) (Fig. 8B).

Media secreted by Ddr2-depleted human ovarian tumor CAFs leads to decreased ovarian tumor cell invasion and migration compared to media secreted by Ddr2-expressing CAFs [25]. We confirmed this result in Matrigel invasion assays in Boyden chambers using two human ovarian tumor cell lines (Tyknu; OVCAR8) and CAF conditioned media (CM) added to the lower well (Fig. 8C, and Supplementary Fig. S8A, B). CM from shDdr2 Arg1^OE^ CAFs rescued this defect (Fig. 8C). CM from Arg1-depleted CAFs did not support ovarian tumor cell invasion through Matrigel (Fig. 8D, and Supplementary Fig. S7D, S8C and S8D). We also performed Matrigel invasion assays using CM from WT CAFs pretreated with the arginase inhibitor, CB1158 [38]. CB1158 was removed from CM prior to invasion assay using a 10 kDa molecular cutoff filter. We observed a dose-response inhibition of tumor cell invasion through Matrigel when conditioned media from CAFs treated with an arginase inhibitor was added (Fig. 8E, and Supplementary Fig. S8E and S8F).

Polyamines are polycationic molecules that can contribute to cellular proliferation and invasion [39, 40]. To determine if DDR2-dependent (Arg1-dependent) polyamine production specifically could contribute to ovarian tumor cell invasion, we added exogenous spermidine or putrescine to CM from Ddr2-depleted CAFs and repeated the Boyden-chamber Matrigel invasion assays. Both polyamines rescued the tumor cell invasion defect of CM from Ddr2-depleted CAFs (Fig. 8F, and Supplementary Fig. S8G and S8H).

In sum, these data indicated that the presence of DDR2 in omental ovarian tumor CAFs controlled polyamine production, likely through DDR2-regulated Arginase-1 production. Moreover, polyamine production by CAFs could support ovarian tumor cell invasion through Matrigel.

### High stromal ARG1 expression in ovarian cancer correlate with poor overall survival

We have previously shown that high stromal expression of DDR2 protein in human ovarian tumors correlates with worse overall survival [22]. Given that DDR2-regulated arginase activity in CAFs affected ovarian cancer tumor collagen production, we asked whether stromal arginase-1 expression correlated with ovarian cancer patient survival. We quantified stromal arginase-1 expression in a human ovarian cancer tumor microarray by immunohistochemistry and correlated stromal arginase-1 protein expression with survival outcomes. Patients with high stromal DDR2 and high stromal arginase-1 expression had median overall survival of 23 months whereas patients with low stromal DDR2 and low stromal arginase-1 had a median overall survival of 171 months (Supplementary Fig. S9A). We then performed a multivariate analysis for DDR2 and arginase-1 controlling for known clinical factors that influence survival. We identified that advanced stage or high stromal arginase-1 were associated with poor survival in ovarian cancer patients (Supplementary Fig. S9B).

## Discussion

DDR2 signaling in multiple preclinical tumor models has been shown to impact tumor progression and metastasis, and high DDR2 expression in human tumor specimens has been associated with worse clinical outcomes [12–14, 16]. We found that although DDR2 is expressed by a subset of tumor cells in human tumor specimens, the majority of DDR2 expression was in CAFs (Fig. 3A, B). DDR2 was not expressed by bone marrow derived myeloid and immune cells (Fig. 3A, B). We have shown in omental CAFs that the presence of DDR2 affects production of collagen and other secreted ECM proteins at the level of their mRNA production or stabilization by mechanisms that have not been explored previously. DDR2 can also impact the cell intrinsic regulation of the collagen binding functions of CAFs that remodel the ECM, such as the collagen fibrillar matrix. Thus, in the absence of DDR2 in CAFs, this results in a tumor ECM that is less permissive for metastatic spread.

Other examples of CAF regulation of collagen protein production include pyrroline-5-carboxylate reductase 1 (PYCR1) and transforming growth factor beta (TGF-β) [41–43]. For example, PYCR1 is highly expressed in CAFs from patients with breast cancer, and this key enzyme is needed for proline synthesis that supports collagen production [41]. TGF- β activated fibroblasts have been found to upregulate production of both proline and glycine to support collagen production in lung cancer [42, 43]. We observed an increase in DDR2 expression in CAFs cultured on polymerized collagen compared to those cultured on plastic (Supplementary Fig. 7E). Thus, it is possible that there could be a feedback loop mechanism of signal amplification where the activation of DDR2 leads to increase in collagen production which further causes DDR2 activation. Further studies are necessary to fully elucidate the role of fibrillar collagen in the amplification of DDR2’s signals.

Much of how fibroblasts support collagen production through upregulation of arginine expression and arginine metabolism is based on work from myofibroblasts in wound healing [44–46], however in CAFs, little is known on how collagen production is metabolically supported. It is expected that CAFs may alter their metabolism accordingly to sustain this ECM production, which is a highly energetic process particularly for structural proteins such as collagens. The collagen protein is composed of glycine, proline and hydroxyproline residues [35]. Proline synthesis is an ATP-consuming process and can be converted from arginine via conversion to ornithine by the enzyme arginase-1 [47]. Ornithine can be further converted to proline by ornithine aminotransferase, and this proline can be used for collagen synthesis.

Our data suggests that CAFs are a major source of arginase activity and L-arginine metabolites in ovarian cancer. This may suggest that CAFs play a significant role in modulating immune cell activity through metabolic availability of arginine and its metabolites. In melanoma, fibroblasts have been found to suppress cytotoxic T lymphocyte activity through increased arginase activity [48, 49]. A prior study showed that tumor cell DDR2 plays a role in response to anti-PD1 therapy [50]. Given our findings on the importance of CAF DDR2, it is possible that fibroblast DDR2 may also contribute to anti-tumor immunity.

This study utilized ovarian mouse and human tumor cell lines as well as CAFs that were cultured from the omentum of patients with metastatic ovarian cancer and mouse CAFs isolated from tumors. We also utilized intraperitoneal models of metastatic colonization similar to Walton et al. and Iyer et al. [23, 24].

A limitation of our study is that we used mice with host global DDR2 knockout for our in vivo tumor burden studies. Prior work has shown that DDR2 plays a role in bone development, lipolysis, and ECM deposition in bone and heart [51–55], so it is possible that DDR2’s role in other cell types contributes to the observed tumor burden phenotype. To clarify the specific role of fibroblast DDR2 in tumor progression, we performed the omental colonization assay and determined that CAF DDR2 and arginase affects the early steps of tumor progression.

In conclusion, our work uncovered that DDR2 signals can regulate collagen protein synthesis and secretion by CAFs from human and mouse ovarian tumors. It does so by controlling the transcription of arginase-1 and thus arginase activity. This transcriptional regulation could occur in a SNAIL1-dependent manner as DDR2 stabilizes SNAIL1 protein levels [28] (Fig. 5A), and SNAIL1 protein was detected at the Arg1 promoter in CAFs. DDR2-dependent arginase activity in CAFs appeared to be critical for collagen deposition in ovarian tumors and could explain how DDR2 regulates tumor ECM fibrillar collagen production and mechanical properties. This work further supports the importance of targeting the tumor microenvironment in cancer progression.

## Materials and methods

### Cell lines and culture

ES2 cells were obtained from NCI and maintained in McCoy’s 5A (modified) medium (Gibco 16600082) supplemented with 10% heat inactivated fetal bovine serum (FBS) and 1% penicillin and streptomycin (Penstrep) (Gibco 15140122). Tyknu cells were maintained in DMEM Medium (Gibco 11965084) supplemented with 10% FBS and 1% Penstrep. Tyknu cells were a gift from Dr. Erinn Rankin. ID8 *Trp53*^−/−^ *Brca2*^−/−^ (ID8TB^−/−^) cells were a gift from Dr. Iain McNeish [23] and were maintained in DMEM with 4%FBS, 1% insulin-transferrin-selenium (ITS) (Gibco 41400045) and 1% Penstrep. KPCA and BPPNM cells were a gift from Dr. Robert Weinberg [24] and were maintained in DMEM with 4%FBS, 1% ITS, 2 ng/ml epidermal growth factor (EGF) (Sigma E9644-.2MG) and 1% Penstrep.

Human CAFs were isolated from the omentum of chemonaïve patients with advanced stage, high-grade serous ovarian or fallopian tube cancer, validated, and maintained as previously described [25, 26]. Patients provided written consent for sample collection and use. Our study was approved by the Washington University Institutional Review Board (IRB 201309050).

Mouse CAFs were isolated from omentum of ID8TB^−/−^ tumor-bearing WT and Ddr2^−/−^ mice as previously published [26]. CAFs were immortalized using SV40 large T virus and negative sort for CD45 and EpCAM was performed. After initial passages, omental CAFs were cultured in DMEM with 10% FBS and 1% Penstrep. Cell lines were maintained at 37 °C in a 5% CO_2_ incubator. STR profiling was performed by IDEXX Bioresearch to authenticate cell lines. Mycoplasma testing was performed using MycoAlert Mycoplasma Detection Kit prior to performing experiments (Lonza LT07-318).

### Mouse tumor burden and in vivo colonization experiments

All animal experiments were performed following the guidelines of the U.S. Public Health Service Policy on Human Care and Use of Laboratory Animal Care and were approved by the Institutional Animal Care and Use Committee at Washington University (Protocol 20-0378). Ddr2^+/−^ mice were bred to produce Ddr2^−/−^ mice and WT (Ddr2^+/+^) littermates [13]. Additional details are in supplemental methods.

### NanoString nCounter mRNA expression profiling

Mouse tumors were collected, and RNA was extracted using RNeasy Mini Kit (Qiagen 74104) and quantified using a Nanodrop (Thermo Fisher). RNA concentration was normalized between samples. RNA was analyzed with the NanoString nCounter Mouse Tumor Signaling 360^TM^ panel (NanoString, Seattle, WA), which contains primers for 20 internal reference control and 760 different tumor signaling-related genes. Additional details are in supplemental methods.

### cDNA preparation and quantitative real-time PCR

RNA was isolated from tumor or cells using the RNeasy Mini Kit (Qiagen 74104) and cDNA was prepared using the SuperScript IV kit (Invitrogen 18091200). SYBR Green PCR Master Mix (Applied Biosystems) and ABI detection system (Applied Biosystems) were used for real-time PCR. Gene expression was quantified using the 2−ΔΔCt method. Additional details are in supplemental methods.

### Immunohistochemical staining and image analysis

Tissues were fixed in formalin for 24 h, embedded in paraffin after graded-ethanol dehydration, and sectioned into 5-μm sections using a microtome. FFPE sections were stained for Hematoxylin & Eosin (Thermo Fisher) and Modified Masson’s Trichrome (Diagnostic Biosystems KT034) according to manufacturer’s instructions. After dewaxing and epitope retrieval, tissues were auto-stained on the Bond Rxm (Leica Biosystems). Staining was visualized using the Bond Polymer Refine Detection alone or in conjunction with Bond Intense R Detection Systems (DS9263, Leica Biosystems). Antibodies used are in Supplemental Table 1. Additional details are in supplemental methods.

### Standard activity and metabolite assays

For all activity assays and metabolite measurements, manufacturer’s guidelines were followed. Experiments were performed in triplicate. Additional details are in supplemental methods.

### Single-cell dissociation, flow cytometry, and single-cell RNA sequencing

Omental ID8TB^−/−^ tumors from WT mice were collected and dissociated in media containing 1 mg/ml collagenase III (Worthington LS004182), 1 mg/ml hyaluronidase (Worthington LS002592), and 0.2 mg/ml DNAse Type IV (Sigma D5025). Mechanical dissociation was performed for 1 min at 500 rpm using the gentlemacs dissociator (Miltenyi Biotec 130-096-427) and enzymatic digestion was performed for 30 min at 37 °C and 150 rpm. Cells were filtered using a 70 μm filter and red blood cells were lysed using RBC lysis buffer (Biolegend 420301). Dead cell removal was performed (Miltenyi Biotec 130-090-101). Cells were resuspended in FACS buffer (PBS, 1 mM EDTA, 4% FBS) and counted. Fc receptors were blocked to reduce non-specific staining and cell surface staining was performed per manufacturer’s recommendation. After cell surface staining was complete, cells were fixed and permeabilized and intracellular staining was performed (Invitrogen 00-5523-00). Antibodies used are listed in Supplemental Table 1. Flow cytometry data was collected on a Cytek Aurora (4L 16UV-16V-14B-8R configuration). After single-cell dissociation and dead cell removal, cells were centrifuged and resuspended in PBS + 0.1%BSA, and final concentration was adjusted to 1000 cells/μl and sent for scRNA processing (10X Genomics). Additional details are in supplemental methods.

### Genetic depletion and overexpression

For all genetic knockdown and overexpression experiments, cells were allowed to undergo antibiotic selection, and polyclonal populations were tested for altered expression levels by immunoblot analysis. Additional details are in supplemental methods.

### Western blot analysis

Protein lysates, collected in 9 mol/l urea and 0.075 mol/l Tris, pH 7.6, were sonicated twice for 15 s and spun down at 10,000 g for 10 min. Protein concentration was quantified using a Bradford protein quantification assay and samples were normalized to 100ug. Lysates were separated by SDS-PAGE and transferred onto nitrocellulose membranes and blocked for 30 min at room temp in 10%milk in 1X TBST. Membranes were incubated overnight in antibodies (see Supplemental Table 1) at 4 °C on a shaker, washed thrice with 1X TBST, and incubated in HRP-conjugated secondary antibody. After three additional washes in TBST, membranes were developed with ECL (Thermo Fisher 34095).

### Polyamine detection by mass spectrometry (MS)

5 × 10^6^ CAFs were cultured at 37 °C in a 5% CO_2_ incubator for 16 h then media was changed to serum free DMEM with 1%Penstrep for 24 h. Serum free conditioned media was collected for downstream MS analysis. Additional details are in supplemental methods.

### Matrigel invasion assay

Boyden chambers (Corning) were filled with 1 mg/ml Matrigel (Corning) and polymerized. CAF conditioned media (CM) was used as the chemoattractant in the lower chamber. 25,000 OVCAR8 cells or 50,000 Tyknu cells were plated in 100 µl media atop the polymerized gel and allowed to invade for 48 h. Polymerized gel was removed from the chambers using a cotton swab. The membrane was fixed, stained, and imaged. Cells were quantified by counting the number of invaded cells per high powered field at 20x. Additional details are in supplemental methods.

### Patient survival and multivariate analysis

Patients provided written consent prior to inclusion in study. Washington University’s Institutional review board gave approval for this study (IRB 201709191). At the time of tumor debulking surgery, samples were collected from patients with advanced stage, high-grade serous ovarian or fallopian tube cancer and used to create an ovarian cancer tissue microarray. Clinical characteristics and survival information were collected from patient charts. Overall survival was determined via Kaplan–Meier analysis using time of death or date of last patient follow-up.

The log-rank test was used for analysis and to differentiate the overall survival between patient groups. Using the ergodicity search (25%~75%), patients were sorted into two groups with low vs. high Arg1 and DDR2 expression and determined the log-rank P-values of overall survival and difference cutoff values. The value where the most significant P-value was determined to be the optimal cutoff level. Survival curves were calculated using the Kaplan–Meier method.

### Arginine tracing experiment

DDR2-expressing and DDR2-depleted CAFs were cultured in media with or without labeled arginine for 72 h, lysed and subjected to mass spectrometry analysis. Additional details are in supplemental methods.

### Chromatin immunoprecipitation

3 × 10^6^ DDR2-expressing and DDR2-depleted CAFs were used for ChIP assay (Abcam ab500) following manufacturer’s protocol. Additional details are in supplemental methods.

### Supplementary information

Supplemental methods

Supplemental Figures and Legends

Supplemental Table 1

Supplemental Table 2

Supplemental Table 3

Supplemental Table 4

Supplemental Table 5

Supplemental Table 6

Supplemental Table 7

## Data Availability

The datasets generated during and/or analyzed during the current study are available in the GEO repository accession code GSE242830.

## References

[1] Levental KR, Yu H, Kass L, Lakins JN, Egeblad M, Erler JT (2009). Matrix crosslinking forces tumor progression by enhancing integrin signaling. Cell.

[2] Calvo F, Ege N, Grande-Garcia A, Hooper S, Jenkins RP, Chaudhry SI (2013). Mechanotransduction and YAP-dependent matrix remodelling is required for the generation and maintenance of cancer-associated fibroblasts. Nat Cell Biol.

[3] Conklin MW, Eickhoff JC, Riching KM, Pehlke CA, Eliceiri KW, Provenzano PP (2011). Aligned collagen is a prognostic signature for survival in human breast carcinoma. Am J Pathol.

[4] Provenzano PP, Eliceiri KW, Campbell JM, Inman DR, White JG, Keely PJ (2006). Collagen reorganization at the tumor-stromal interface facilitates local invasion. BMC Med.

[5] Bayer SVH, Grither WR, Brenot A, Hwang PY, Barcus CE, Ernst M (2019). DDR2 controls breast tumor stiffness and metastasis by regulating integrin mediated mechanotransduction in CAFs. eLife.

[6] Provenzano PP, Inman DR, Eliceiri KW, Knittel JG, Yan L, Rueden CT (2008). Collagen density promotes mammary tumor initiation and progression. BMC Med.

[7] Kalluri R, Zeisberg M (2006). Fibroblasts in cancer. Nat Rev Cancer.

[8] Gilkes DM, Semenza GL, Wirtz D (2014). Hypoxia and the extracellular matrix: drivers of tumour metastasis. Nat Rev Cancer.

[9] Cho A, Howell VM, Colvin EK (2015). The extracellular matrix in epithelial ovarian cancer - a piece of a puzzle. Front Oncol.

[10] Kenny HA, Kaur S, Coussens LM, Lengyel E (2008). The initial steps of ovarian cancer cell metastasis are mediated by MMP-2 cleavage of vitronectin and fibronectin. J Clin Investig.

[11] Kenny HA, Chiang CY, White EA, Schryver EM, Habis M, Romero IL (2014). Mesothelial cells promote early ovarian cancer metastasis through fibronectin secretion. J Clin Invest.

[12] Chua HH, Yeh TH, Wang YP, Huang YT, Sheen TS, Lo YC (2008). Upregulation of discoidin domain receptor 2 in nasopharyngeal carcinoma. Head Neck.

[13] Corsa CA, Brenot A, Grither WR, Van Hove S, Loza AJ, Zhang K (2016). The action of discoidin domain receptor 2 in basal tumor cells and stromal cancer-associated fibroblasts is critical for breast cancer metastasis. Cell Rep.

[14] Hammerman PS, Sos ML, Ramos AH, Xu C, Dutt A, Zhou W (2011). Mutations in the DDR2 kinase gene identify a novel therapeutic target in squamous cell lung cancer. Cancer Discov.

[15] Ren T, Zhang W, Liu X, Zhao H, Zhang J, Zhang J (2014). Discoidin domain receptor 2 (DDR2) promotes breast cancer cell metastasis and the mechanism implicates epithelial-mesenchymal transition programme under hypoxia. J Pathol.

[16] Xie B, Lin W, Ye J, Wang X, Zhang B, Xiong S (2015). DDR2 facilitates hepatocellular carcinoma invasion and metastasis via activating ERK signaling and stabilizing SNAIL1. J Exp Clin Cancer Res.

[17] Grither WR, Divine LM, Meller EH, Wilke DJ, Desai RA, Loza AJ (2018). TWIST1 induces expression of discoidin domain receptor 2 to promote ovarian cancer metastasis. Oncogene.

[18] Grzywa TM, Sosnowska A, Matryba P, Rydzynska Z, Jasinski M, Nowis D (2020). Myeloid cell-derived arginase in cancer immune response. Front Immunol.

[19] Chang CI, Liao JC, Kuo L (2001). Macrophage arginase promotes tumor cell growth and suppresses nitric oxide-mediated tumor cytotoxicity. Cancer Res.

[20] Munder M, Engelhardt M, Knies D, Medenhoff S, Wabnitz G, Luckner-Minden C (2013). Cytotoxicity of tumor antigen specific human T cells is unimpaired by arginine depletion. PLoS ONE.

[21] Kumar V, Patel S, Tcyganov E, Gabrilovich DI (2016). The nature of myeloid-derived suppressor cells in the tumor microenvironment. Trends Immunol.

[22] Schab AM, Greenwade MM, Stock E, Lomonosova E, Cho K, Grither WR, et al. Stromal DDR2 Promotes Ovarian Cancer Metastasis through Regulation of Metabolism and Secretion of Extracellular Matrix Proteins. Mol Cancer Res. 2023;21:1234–48. 10.1158/1541-7786.MCR-23-0347 PMC10832402 37527178

[23] Walton J, Blagih J, Ennis D, Leung E, Dowson S, Farquharson M (2016). CRISPR/Cas9-mediated Trp53 and Brca2 knockout to generate improved murine models of ovarian high-grade serous carcinoma. Cancer Res.

[24] Iyer S, Zhang S, Yucel S, Horn H, Smith SG, Reinhardt F (2021). Genetically defined syngeneic mouse models of ovarian cancer as tools for the discovery of combination immunotherapy. Cancer Discov.

[25] Akinjiyan FA, Dave RM, Alpert E, Longmore GD, Fuh KC (2022). DDR2 expression in cancer-associated fibroblasts promotes ovarian cancer tumor invasion and metastasis through periostin-ITGB1. Cancers.

[26] Kenny HA, Krausz T, Yamada SD, Lengyel E (2007). Use of a novel 3D culture model to elucidate the role of mesothelial cells, fibroblasts and extra-cellular matrices on adhesion and invasion of ovarian cancer cells to the omentum. Int J Cancer.

[27] Wang YL, Zhao XM, Shuai ZF, Li CY, Bai QY, Yu XW (2015). Snail promotes epithelial-mesenchymal transition and invasiveness in human ovarian cancer cells. Int J Clin Exp Med.

[28] Zhang K, Corsa CA, Ponik SM, Prior JL, Piwnica-Worms D, Eliceiri KW (2013). The collagen receptor discoidin domain receptor 2 stabilizes SNAIL1 to facilitate breast cancer metastasis. Nat Cell Biol.

[29] Cano A, Pérez-Moreno MA, Rodrigo I, Locascio A, Blanco MJ, del Barrio MG (2000). The transcription factor Snail controls epithelial–mesenchymal transitions by repressing E-cadherin expression. Nat Cell Biol.

[30] Stanisavljevic J, Porta-de-la-Riva M, Batlle R, de Herreros AG, Baulida J (2011). The p65 subunit of NF-κB and PARP1 assist Snail1 in activating fibronectin transcription. J Cell Sci.

[31] Hsu DS, Wang HJ, Tai SK, Chou CH, Hsieh CH, Chiu PH (2014). Acetylation of snail modulates the cytokinome of cancer cells to enhance the recruitment of macrophages. Cancer Cell.

[32] Pegg AE, Williams-Ashman HG (1968). Biosynthesis of putrescine in the prostate gland of the rat. Biochem J.

[33] Pegg AE, Williams-Ashman HG (1969). On the role of S-adenosyl-L-methionine in the biosynthesis of spermidine by rat prostate. J Biol Chem.

[34] Tabor H, Tabor CW (1964). Spermidine, spermine, and related amines. Pharm Rev.

[35] Li P, Wu G (2018). Roles of dietary glycine, proline, and hydroxyproline in collagen synthesis and animal growth. Amino Acids.

[36] Kivirikko KI, Pihlajaniemi T (2009). Collagen hydroxylases and the protein disulfide isomerase subunit of prolyl 4-hydroxylases. Adv Enzymol Relat Areas Mol Biol.

[37] Seifter E, Rettura G, Barbul A, Levenson SM (1978). Arginine: an essential amino acid for injured rats. Surgery.

[38] Steggerda SM, Bennett MK, Chen J, Emberley E, Huang T, Janes JR (2017). Inhibition of arginase by CB-1158 blocks myeloid cell-mediated immune suppression in the tumor microenvironment. J Immunother Cancer.

[39] Mandal S, Mandal A, Johansson HE, Orjalo AV, Park MH (2013). Depletion of cellular polyamines, spermidine and spermine, causes a total arrest in translation and growth in mammalian cells. Proc Natl Acad Sci.

[40] Kubota S, Kiyosawa H, Nomura Y, Yamada T, Seyama Y (1997). Ornithine decarboxylase overexpression in mouse 10T12 fibroblasts: cellular transformation and invasion. J Natl Cancer Inst.

[41] Kay EJ, Paterson K, Riera-Domingo C, Sumpton D, Däbritz JHM, Tardito S (2022). Cancer-associated fibroblasts require proline synthesis by PYCR1 for the deposition of pro-tumorigenic extracellular matrix. Nat Metab.

[42] Schwörer S, Berisa M, Violante S, Qin W, Zhu J, Hendrickson RC (2020). Proline biosynthesis is a vent for TGFβ-induced mitochondrial redox stress. EMBO J.

[43] Nigdelioglu R, Hamanaka RB, Meliton AY, O’Leary E, Witt LJ, Cho T (2016). Transforming growth factor (TGF)-β promotes de Novo serine synthesis for collagen production. J Biol Chem.

[44] Foster DS, Jones RE, Ransom RC, Longaker MT, Norton JA. The evolving relationship of wound healing and tumor stroma. JCI Insight. 2018;3. https://insight.jci.org/articles/view/99911, https://pubmed.ncbi.nlm.nih.gov/30232274/. 10.1172/jci.insight.99911 PMC6237224 30232274

[45] Witte MB, Barbul A, Schick MA, Vogt N, Becker HD (2002). Upregulation of arginase expression in wound-derived fibroblasts. J Surg Res.

[46] Campbell L, Saville CR, Murray PJ, Cruickshank SM, Hardman MJ (2013). Local arginase 1 activity is required for cutaneous wound healing. J Invest Dermatol.

[47] Kay EJ, Koulouras G, Zanivan S (2021). Regulation of extracellular matrix production in activated fibroblasts: roles of amino acid metabolism in collagen synthesis. Front Oncol.

[48] Ino Y, Yamazaki-Itoh R, Oguro S, Shimada K, Kosuge T, Zavada J (2013). Arginase II expressed in cancer-associated fibroblasts indicates tissue hypoxia and predicts poor outcome in patients with pancreatic cancer. PLoS ONE.

[49] Érsek B, Silló P, Cakir U, Molnár V, Bencsik A, Mayer B (2021). Melanoma-associated fibroblasts impair CD8+ T cell function and modify expression of immune checkpoint regulators via increased arginase activity. Cell Mol Life Sci.

[50] Tu MM, Lee FYF, Jones RT, Kimball AK, Saravia E, Graziano RF (2019). Targeting DDR2 enhances tumor response to anti–PD-1 immunotherapy. Sci Adv.

[51] Yang X, Li J, Zhao L, Chen Y, Cui Z, Xu T (2022). Targeting adipocytic discoidin domain receptor 2 impedes fat gain while increasing bone mass. Cell Death Differ.

[52] Lin KL, Chou CH, Hsieh SC, Hwa SY, Lee MT, Wang FF (2010). Transcriptional upregulation of DDR2 by ATF4 facilitates osteoblastic differentiation through p38 MAPK-mediated Runx2 activation. J Bone Min Res.

[53] Mohamed FF, Ge C, Cowling RT, Lucas D, Hallett SA, Ono N (2022). The collagen receptor, discoidin domain receptor 2, functions in Gli1-positive skeletal progenitors and chondrocytes to control bone development. Bone Res.

[54] Labrador JP, Azcoitia V, Tuckermann J, Lin C, Olaso E, Mañes S (2001). The collagen receptor DDR2 regulates proliferation and its elimination leads to dwarfism. EMBO Rep.

[55] Pagani CA, Bancroft AC, Tower RJ, Livingston N, Sun Y, Hong JY (2022). Discoidin domain receptor 2 regulates aberrant mesenchymal lineage cell fate and matrix organization. Sci Adv.

