# Supplementary material for: DDR2-regulated arginase activity in ovarian cancer-associated fibroblasts promotes collagen production and tumor progression
Source: Oncogene. Author manuscript; Available in PMC 2024 Feb 16. (PMC10786713; doi:10.1038/s41388-023-02884-3)
Supplement: Supplementary Figures 1-9 [file NIHMS1947626-supplement-Supplementary_Figures_1-9.pptx]

## Slide 1
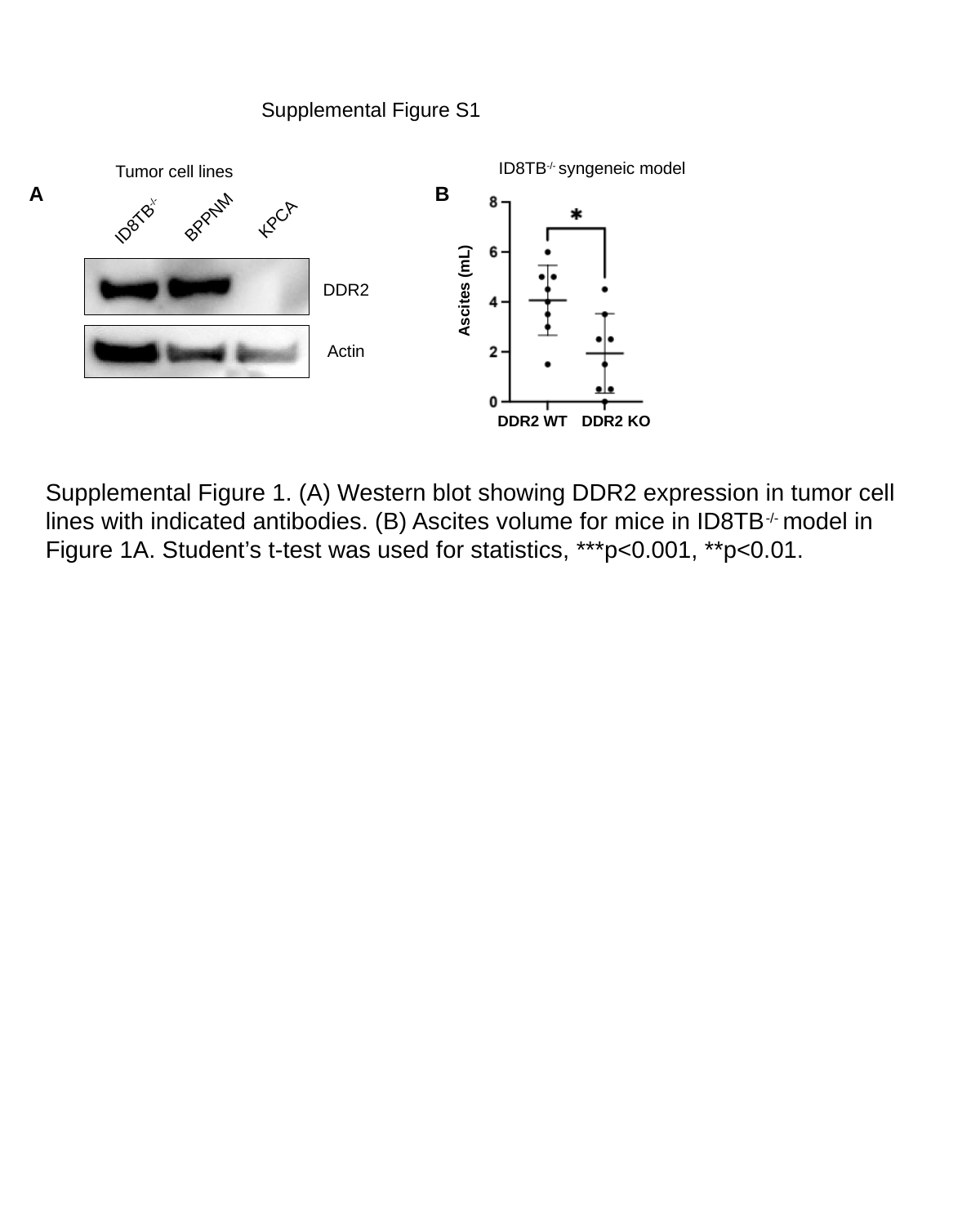

Supplemental Figure S1
ID8TB-/- syngeneic model
Tumor cell lines
A
B
KPCA
BPPNM
ID8TB-/-
DDR2
Actin
Ascites (mL)
DDR2 WT
DDR2 KO
Supplemental Figure 1. (A) Western blot showing DDR2 expression in tumor cell lines with indicated antibodies. (B) Ascites volume for mice in ID8TB-/- model in Figure 1A. Student’s t-test was used for statistics, ***p<0.001, **p<0.01.

## Slide 2
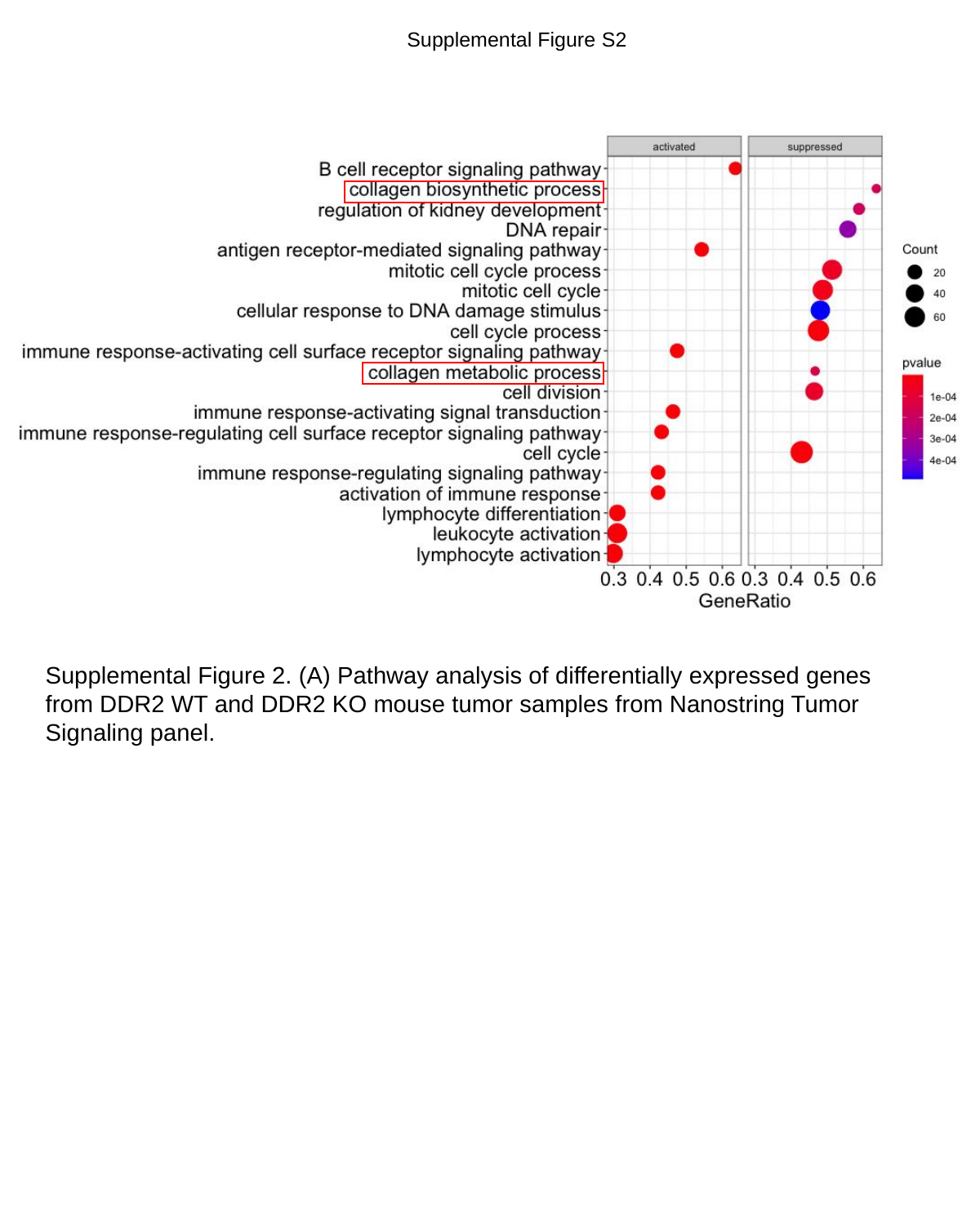

Supplemental Figure S2
Supplemental Figure 2. (A) Pathway analysis of differentially expressed genes from DDR2 WT and DDR2 KO mouse tumor samples from Nanostring Tumor Signaling panel.

## Slide 3
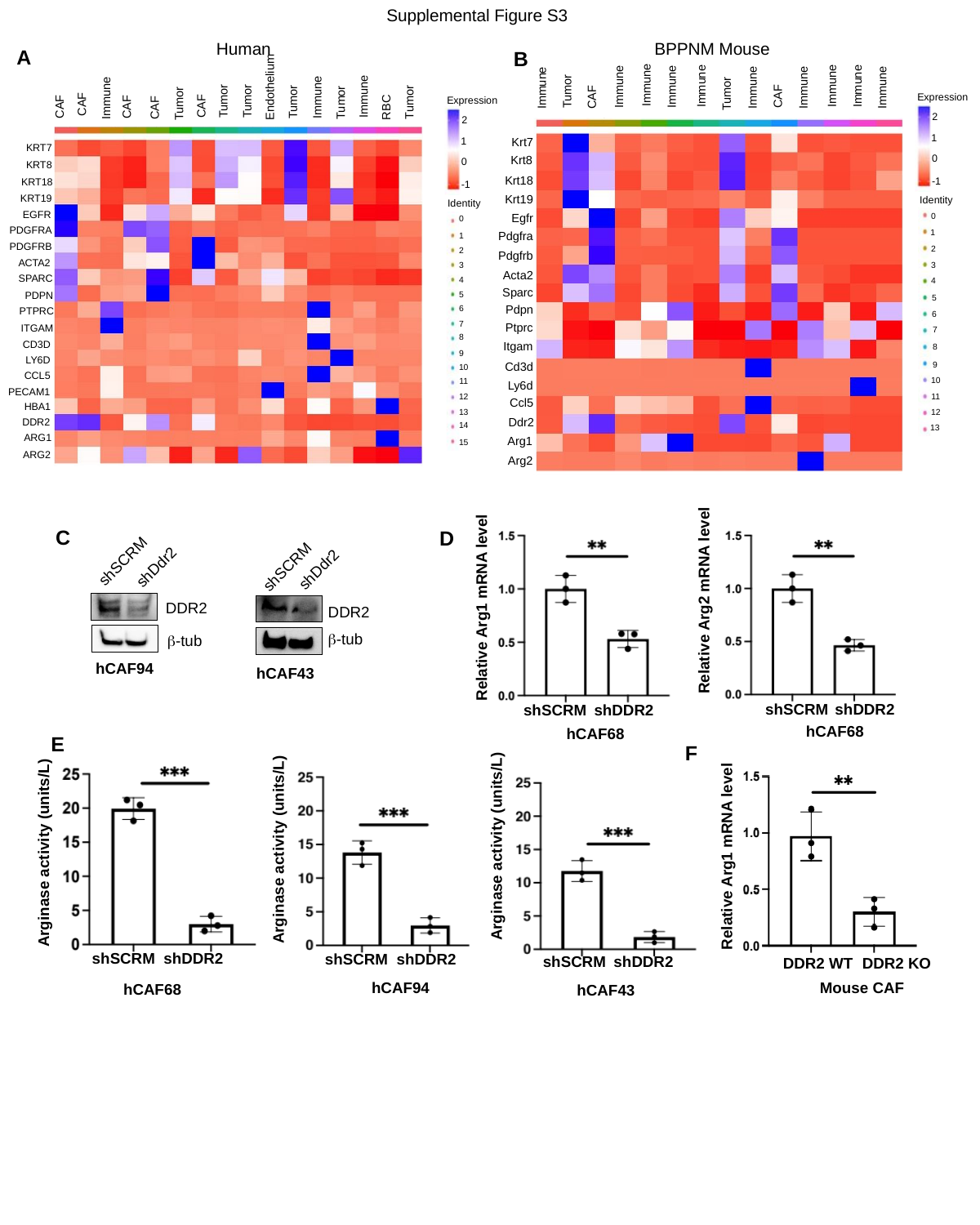

Supplemental Figure S3
Human
BPPNM Mouse
A
B
Endothelium
Immune
Immune
Immune
Immune
Immune
Immune
Immune
Immune
Immune
Immune
Tumor
Immune
Immune
Immune
Tumor
Tumor
Tumor
Tumor
Tumor
CAF
Tumor
Expression
CAF
Tumor
Expression
CAF
CAF
CAF
RBC
CAF
CAF
2
2
1
1
Krt7
KRT7
0
Krt8
0
KRT8
Krt18
-1
KRT18
-1
KRT19
Krt19
Identity
Identity
EGFR
Egfr
0
0
PDGFRA
1
Pdgfra
1
PDGFRB
2
2
Pdgfrb
ACTA2
3
3
Acta2
SPARC
4
4
Sparc
PDPN
5
5
Pdpn
6
PTPRC
6
7
Ptprc
ITGAM
7
8
CD3D
Itgam
8
9
LY6D
Cd3d
9
10
CCL5
10
11
Ly6d
PECAM1
11
12
Ccl5
HBA1
13
12
Ddr2
DDR2
14
13
ARG1
Arg1
15
ARG2
Arg2
Relative Arg2 mRNA level
Relative Arg1 mRNA level
C
D
shSCRM
shDdr2
DDR2
b-tub
hCAF94
shSCRM
shDdr2
DDR2
b-tub
hCAF43
shSCRM
shDDR2
shSCRM
shDDR2
hCAF68
hCAF68
E
F
Arginase activity (units/L)
Arginase activity (units/L)
Arginase activity (units/L)
Relative Arg1 mRNA level
shSCRM
shDDR2
shSCRM
shDDR2
shSCRM
shDDR2
DDR2 WT
DDR2 KO
hCAF94
Mouse CAF
hCAF68
hCAF43

## Slide 4
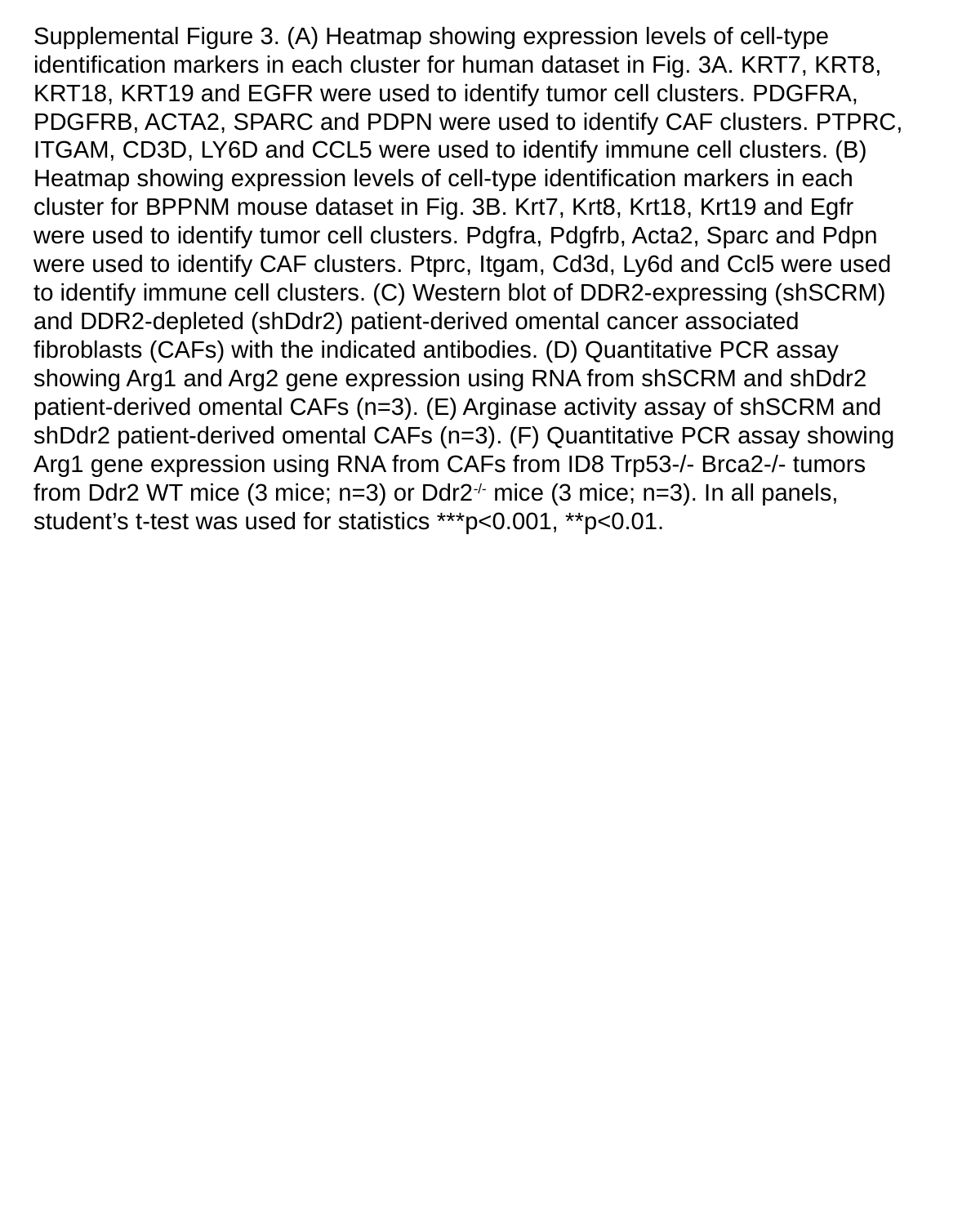

Supplemental Figure 3. (A) Heatmap showing expression levels of cell-type identification markers in each cluster for human dataset in Fig. 3A. KRT7, KRT8, KRT18, KRT19 and EGFR were used to identify tumor cell clusters. PDGFRA, PDGFRB, ACTA2, SPARC and PDPN were used to identify CAF clusters. PTPRC, ITGAM, CD3D, LY6D and CCL5 were used to identify immune cell clusters. (B) Heatmap showing expression levels of cell-type identification markers in each cluster for BPPNM mouse dataset in Fig. 3B. Krt7, Krt8, Krt18, Krt19 and Egfr were used to identify tumor cell clusters. Pdgfra, Pdgfrb, Acta2, Sparc and Pdpn were used to identify CAF clusters. Ptprc, Itgam, Cd3d, Ly6d and Ccl5 were used to identify immune cell clusters. (C) Western blot of DDR2-expressing (shSCRM) and DDR2-depleted (shDdr2) patient-derived omental cancer associated fibroblasts (CAFs) with the indicated antibodies. (D) Quantitative PCR assay showing Arg1 and Arg2 gene expression using RNA from shSCRM and shDdr2 patient-derived omental CAFs (n=3). (E) Arginase activity assay of shSCRM and shDdr2 patient-derived omental CAFs (n=3). (F) Quantitative PCR assay showing Arg1 gene expression using RNA from CAFs from ID8 Trp53-/- Brca2-/- tumors from Ddr2 WT mice (3 mice; n=3) or Ddr2-/- mice (3 mice; n=3). In all panels, student’s t-test was used for statistics ***p<0.001, **p<0.01.

## Slide 5
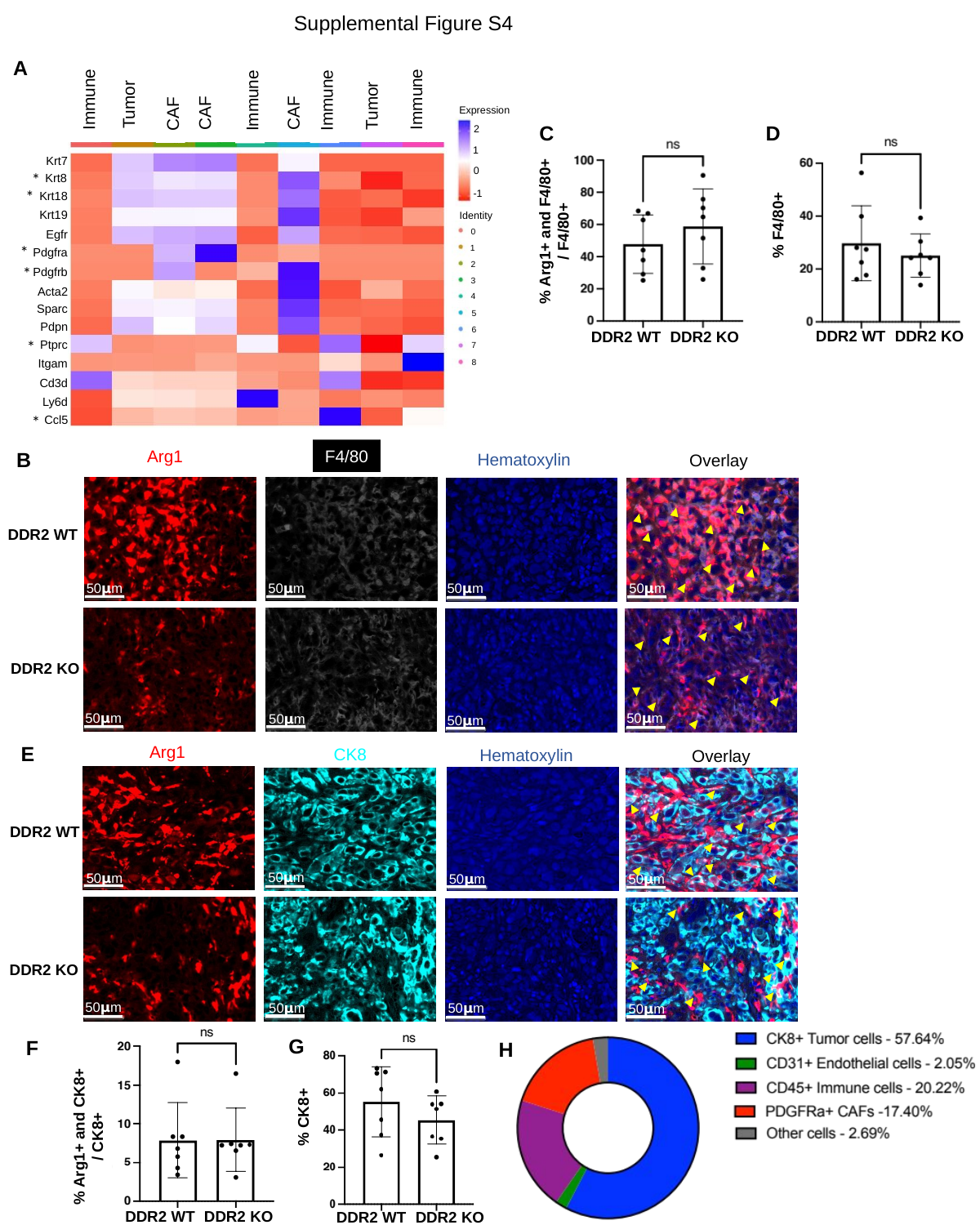

Supplemental Figure S4
A
Immune
Immune
Immune
Immune
Tumor
Tumor
Expression
CAF
CAF
CAF
C
D
2
1
Krt7
% Arg1+ and F4/80+
/ F4/80+
0
*
Krt8
*
-1
Krt18
% F4/80+
Krt19
Identity
0
Egfr
*
1
Pdgfra
2
*
Pdgfrb
3
Acta2
4
Sparc
5
Pdpn
6
DDR2 WT
DDR2 KO
DDR2 WT
DDR2 KO
*
Ptprc
7
Itgam
8
Cd3d
Ly6d
*
Ccl5
Arg1
F4/80
B
Hematoxylin
Overlay
DDR2 WT
50𝝻m
50𝝻m
50𝝻m
50𝝻m
DDR2 KO
50𝝻m
50𝝻m
50𝝻m
50𝝻m
E
Arg1
CK8
Hematoxylin
Overlay
DDR2 WT
50𝝻m
50𝝻m
50𝝻m
50𝝻m
DDR2 KO
50𝝻m
50𝝻m
50𝝻m
50𝝻m
G
F
H
% Arg1+ and CK8+
/ CK8+
% CK8+
DDR2 WT
DDR2 KO
DDR2 WT
DDR2 KO

## Slide 6
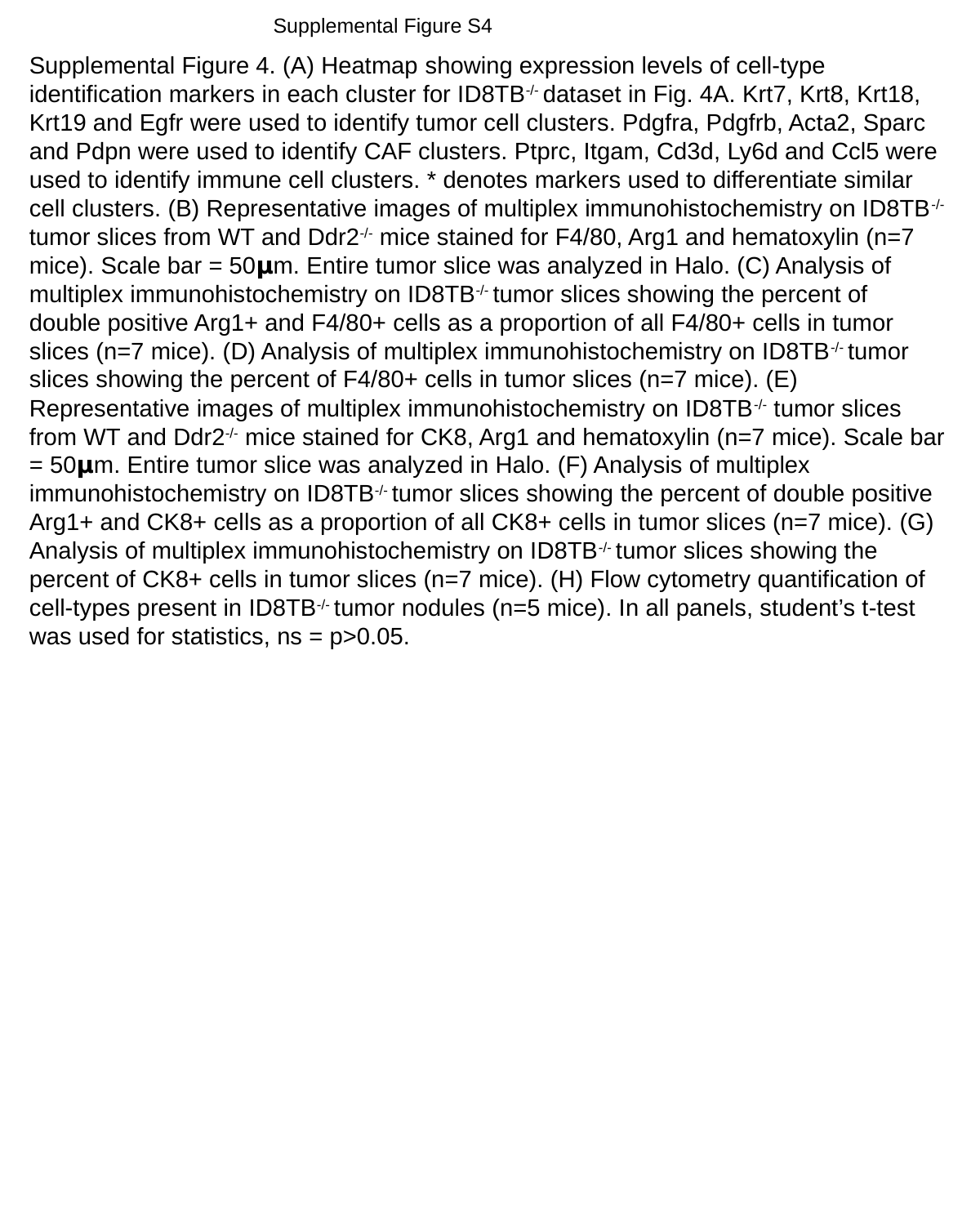

Supplemental Figure S4
Supplemental Figure 4. (A) Heatmap showing expression levels of cell-type identification markers in each cluster for ID8TB-/- dataset in Fig. 4A. Krt7, Krt8, Krt18, Krt19 and Egfr were used to identify tumor cell clusters. Pdgfra, Pdgfrb, Acta2, Sparc and Pdpn were used to identify CAF clusters. Ptprc, Itgam, Cd3d, Ly6d and Ccl5 were used to identify immune cell clusters. * denotes markers used to differentiate similar cell clusters. (B) Representative images of multiplex immunohistochemistry on ID8TB-/- tumor slices from WT and Ddr2-/- mice stained for F4/80, Arg1 and hematoxylin (n=7 mice). Scale bar = 50𝝻m. Entire tumor slice was analyzed in Halo. (C) Analysis of multiplex immunohistochemistry on ID8TB-/- tumor slices showing the percent of double positive Arg1+ and F4/80+ cells as a proportion of all F4/80+ cells in tumor slices (n=7 mice). (D) Analysis of multiplex immunohistochemistry on ID8TB-/- tumor slices showing the percent of F4/80+ cells in tumor slices (n=7 mice). (E) Representative images of multiplex immunohistochemistry on ID8TB-/- tumor slices from WT and Ddr2-/- mice stained for CK8, Arg1 and hematoxylin (n=7 mice). Scale bar = 50𝝻m. Entire tumor slice was analyzed in Halo. (F) Analysis of multiplex immunohistochemistry on ID8TB-/- tumor slices showing the percent of double positive Arg1+ and CK8+ cells as a proportion of all CK8+ cells in tumor slices (n=7 mice). (G) Analysis of multiplex immunohistochemistry on ID8TB-/- tumor slices showing the percent of CK8+ cells in tumor slices (n=7 mice). (H) Flow cytometry quantification of cell-types present in ID8TB-/- tumor nodules (n=5 mice). In all panels, student’s t-test was used for statistics, ns = p>0.05.

## Slide 7
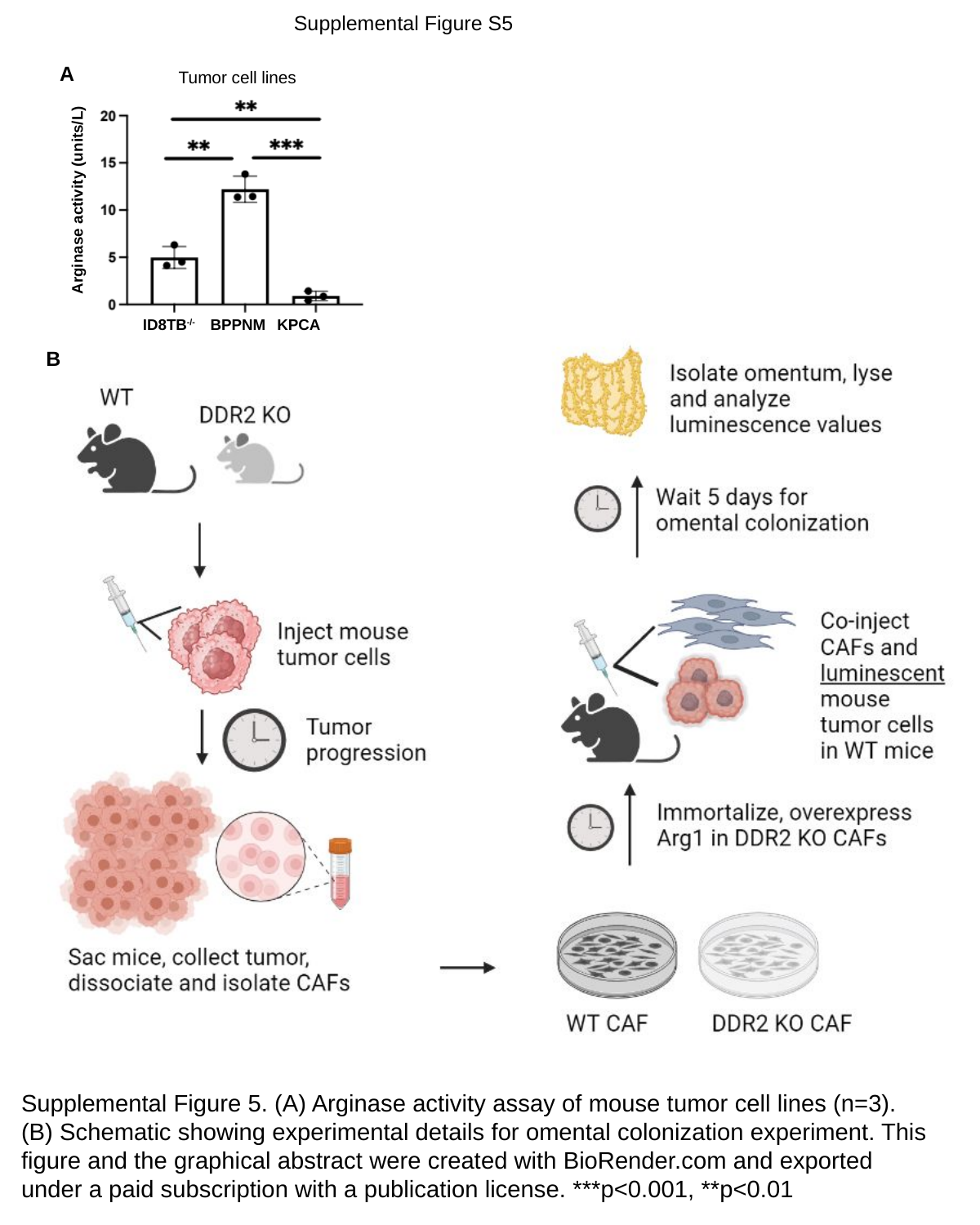

Supplemental Figure S5
A
Tumor cell lines
Arginase activity (units/L)
ID8TB-/-
BPPNM
KPCA
B
Supplemental Figure 5. (A) Arginase activity assay of mouse tumor cell lines (n=3). (B) Schematic showing experimental details for omental colonization experiment. This figure and the graphical abstract were created with BioRender.com and exported under a paid subscription with a publication license. ***p<0.001, **p<0.01

## Slide 8
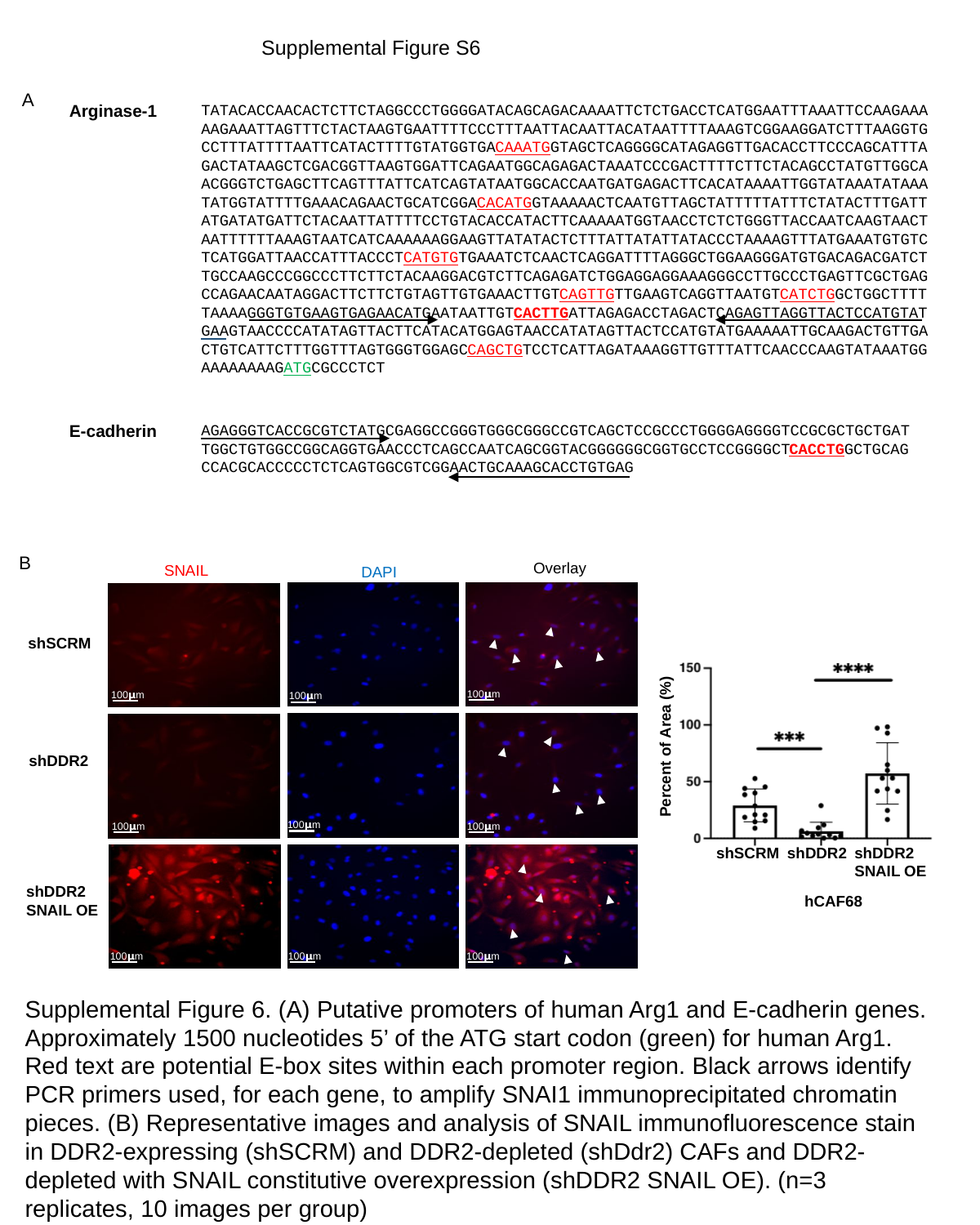

Supplemental Figure S6
A
Arginase-1
TATACACCAACACTCTTCTAGGCCCTGGGGATACAGCAGACAAAATTCTCTGACCTCATGGAATTTAAATTCCAAGAAAAAGAAATTAGTTTCTACTAAGTGAATTTTCCCTTTAATTACAATTACATAATTTTAAAGTCGGAAGGATCTTTAAGGTGCCTTTATTTTAATTCATACTTTTGTATGGTGACAAATGGTAGCTCAGGGGCATAGAGGTTGACACCTTCCCAGCATTTAGACTATAAGCTCGACGGTTAAGTGGATTCAGAATGGCAGAGACTAAATCCCGACTTTTCTTCTACAGCCTATGTTGGCAACGGGTCTGAGCTTCAGTTTATTCATCAGTATAATGGCACCAATGATGAGACTTCACATAAAATTGGTATAAATATAAATATGGTATTTTGAAACAGAACTGCATCGGACACATGGTAAAAACTCAATGTTAGCTATTTTTATTTCTATACTTTGATTATGATATGATTCTACAATTATTTTCCTGTACACCATACTTCAAAAATGGTAACCTCTCTGGGTTACCAATCAAGTAACTAATTTTTTAAAGTAATCATCAAAAAAGGAAGTTATATACTCTTTATTATATTATACCCTAAAAGTTTATGAAATGTGTCTCATGGATTAACCATTTACCCTCATGTGTGAAATCTCAACTCAGGATTTTAGGGCTGGAAGGGATGTGACAGACGATCTTGCCAAGCCCGGCCCTTCTTCTACAAGGACGTCTTCAGAGATCTGGAGGAGGAAAGGGCCTTGCCCTGAGTTCGCTGAGCCAGAACAATAGGACTTCTTCTGTAGTTGTGAAACTTGTCAGTTGTTGAAGTCAGGTTAATGTCATCTGGCTGGCTTTTTAAAAGGGTGTGAAGTGAGAACATGAATAATTGTCACTTGATTAGAGACCTAGACTCAGAGTTAGGTTACTCCATGTATGAAGTAACCCCATATAGTTACTTCATACATGGAGTAACCATATAGTTACTCCATGTATGAAAAATTGCAAGACTGTTGACTGTCATTCTTTGGTTTAGTGGGTGGAGCCAGCTGTCCTCATTAGATAAAGGTTGTTTATTCAACCCAAGTATAAATGGAAAAAAAAGATGCGCCCTCT
E-cadherin
AGAGGGTCACCGCGTCTATGCGAGGCCGGGTGGGCGGGCCGTCAGCTCCGCCCTGGGGAGGGGTCCGCGCTGCTGATTGGCTGTGGCCGGCAGGTGAACCCTCAGCCAATCAGCGGTACGGGGGGCGGTGCCTCCGGGGCTCACCTGGCTGCAGCCACGCACCCCCTCTCAGTGGCGTCGGAACTGCAAAGCACCTGTGAG
B
Overlay
SNAIL
DAPI
100𝝻m
100𝝻m
100𝝻m
100𝝻m
100𝝻m
100𝝻m
100𝝻m
100𝝻m
100𝝻m
shSCRM
Percent of Area (%)
shDDR2
shDDR2
SNAIL OE
shSCRM
shDDR2
shDDR2
SNAIL OE
hCAF68
Supplemental Figure 6. (A) Putative promoters of human Arg1 and E-cadherin genes.
Approximately 1500 nucleotides 5’ of the ATG start codon (green) for human Arg1. Red text are potential E-box sites within each promoter region. Black arrows identify PCR primers used, for each gene, to amplify SNAI1 immunoprecipitated chromatin pieces. (B) Representative images and analysis of SNAIL immunofluorescence stain in DDR2-expressing (shSCRM) and DDR2-depleted (shDdr2) CAFs and DDR2-depleted with SNAIL constitutive overexpression (shDDR2 SNAIL OE). (n=3 replicates, 10 images per group)

## Slide 9
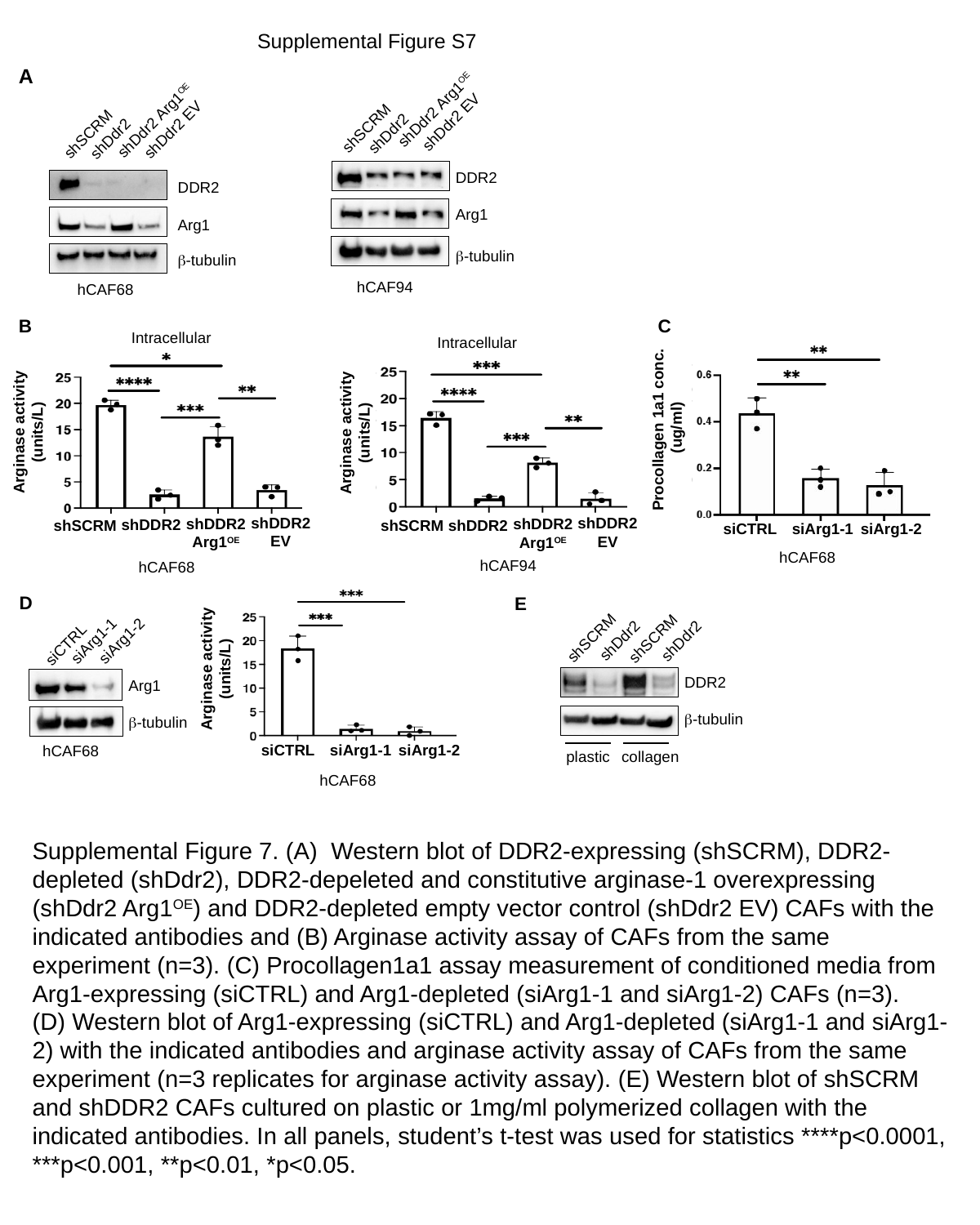

Supplemental Figure S7
A
shDdr2 Arg1OE
shDdr2 Arg1OE
shDdr2 EV
shSCRM
shDdr2 EV
shDdr2
shSCRM
shDdr2
DDR2
DDR2
Arg1
Arg1
b-tubulin
b-tubulin
hCAF94
hCAF68
B
C
Intracellular
Intracellular
Procollagen 1a1 conc.
(ug/ml)
Arginase activity
(units/L)
Arginase activity
(units/L)
shDDR2
EV
shDDR2
EV
shDDR2
Arg1OE
shDDR2
Arg1OE
shDDR2
shSCRM
shDDR2
shSCRM
siCTRL
siArg1-1
siArg1-2
hCAF68
hCAF94
hCAF68
D
E
Arginase activity
(units/L)
shSCRM
shSCRM
siArg1-1
siArg1-2
shDdr2
shDdr2
siCTRL
DDR2
Arg1
b-tubulin
b-tubulin
siCTRL
siArg1-1
siArg1-2
hCAF68
plastic
collagen
hCAF68
Supplemental Figure 7. (A) Western blot of DDR2-expressing (shSCRM), DDR2-depleted (shDdr2), DDR2-depeleted and constitutive arginase-1 overexpressing (shDdr2 Arg1OE) and DDR2-depleted empty vector control (shDdr2 EV) CAFs with the indicated antibodies and (B) Arginase activity assay of CAFs from the same experiment (n=3). (C) Procollagen1a1 assay measurement of conditioned media from Arg1-expressing (siCTRL) and Arg1-depleted (siArg1-1 and siArg1-2) CAFs (n=3).
(D) Western blot of Arg1-expressing (siCTRL) and Arg1-depleted (siArg1-1 and siArg1-2) with the indicated antibodies and arginase activity assay of CAFs from the same experiment (n=3 replicates for arginase activity assay). (E) Western blot of shSCRM and shDDR2 CAFs cultured on plastic or 1mg/ml polymerized collagen with the indicated antibodies. In all panels, student’s t-test was used for statistics ****p<0.0001, ***p<0.001, **p<0.01, *p<0.05.

## Slide 10
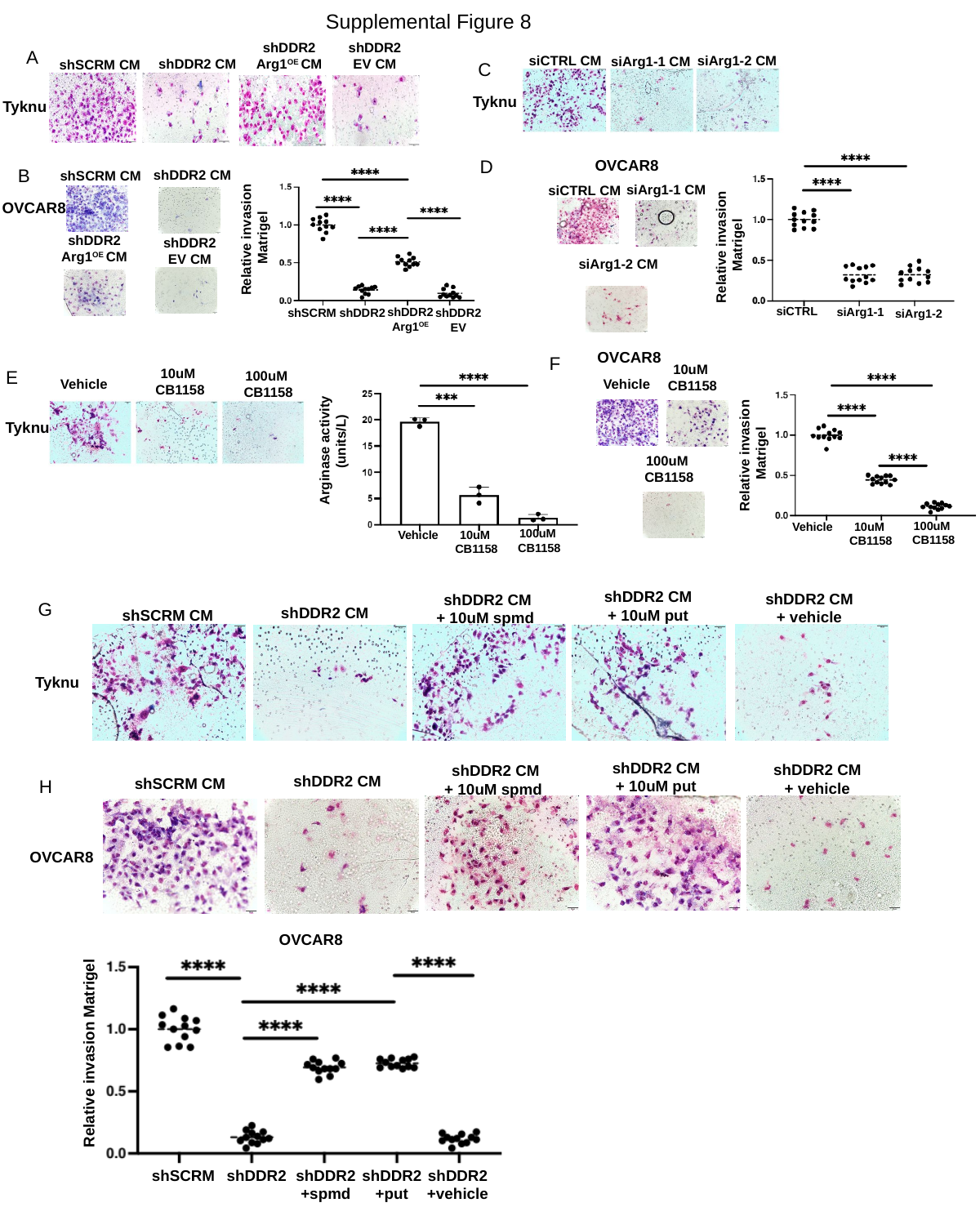

Supplemental Figure 8
shDDR2
Arg1OE CM
shDDR2
EV CM
A
siArg1-2 CM
siCTRL CM
siArg1-1 CM
shDDR2 CM
shSCRM CM
C
Tyknu
Tyknu
D
OVCAR8
B
shDDR2 CM
shSCRM CM
shDDR2
Arg1OE CM
shDDR2
EV CM
siArg1-1 CM
siCTRL CM
Relative invasion
 Matrigel
Relative invasion
 Matrigel
OVCAR8
siArg1-2 CM
siCTRL
shDDR2
Arg1OE
siArg1-1
shSCRM
shDDR2
shDDR2
EV
siArg1-2
OVCAR8
F
10uM
CB1158
E
10uM
CB1158
100uM
CB1158
Vehicle
Arginase activity
(units/L)
Vehicle
Relative invasion
 Matrigel
Tyknu
100uM
CB1158
100uM
CB1158
Vehicle
10uM
CB1158
100uM
CB1158
Vehicle
10uM
CB1158
shDDR2 CM
+ 10uM put
shDDR2 CM
+ vehicle
shDDR2 CM
+ 10uM spmd
shDDR2 CM
shSCRM CM
G
Tyknu
shDDR2 CM
+ 10uM put
shDDR2 CM
+ vehicle
shDDR2 CM
+ 10uM spmd
shDDR2 CM
shSCRM CM
H
OVCAR8
OVCAR8
Relative invasion Matrigel
shDDR2
shDDR2
+vehicle
shDDR2
+spmd
shDDR2
+put
shSCRM

## Slide 11
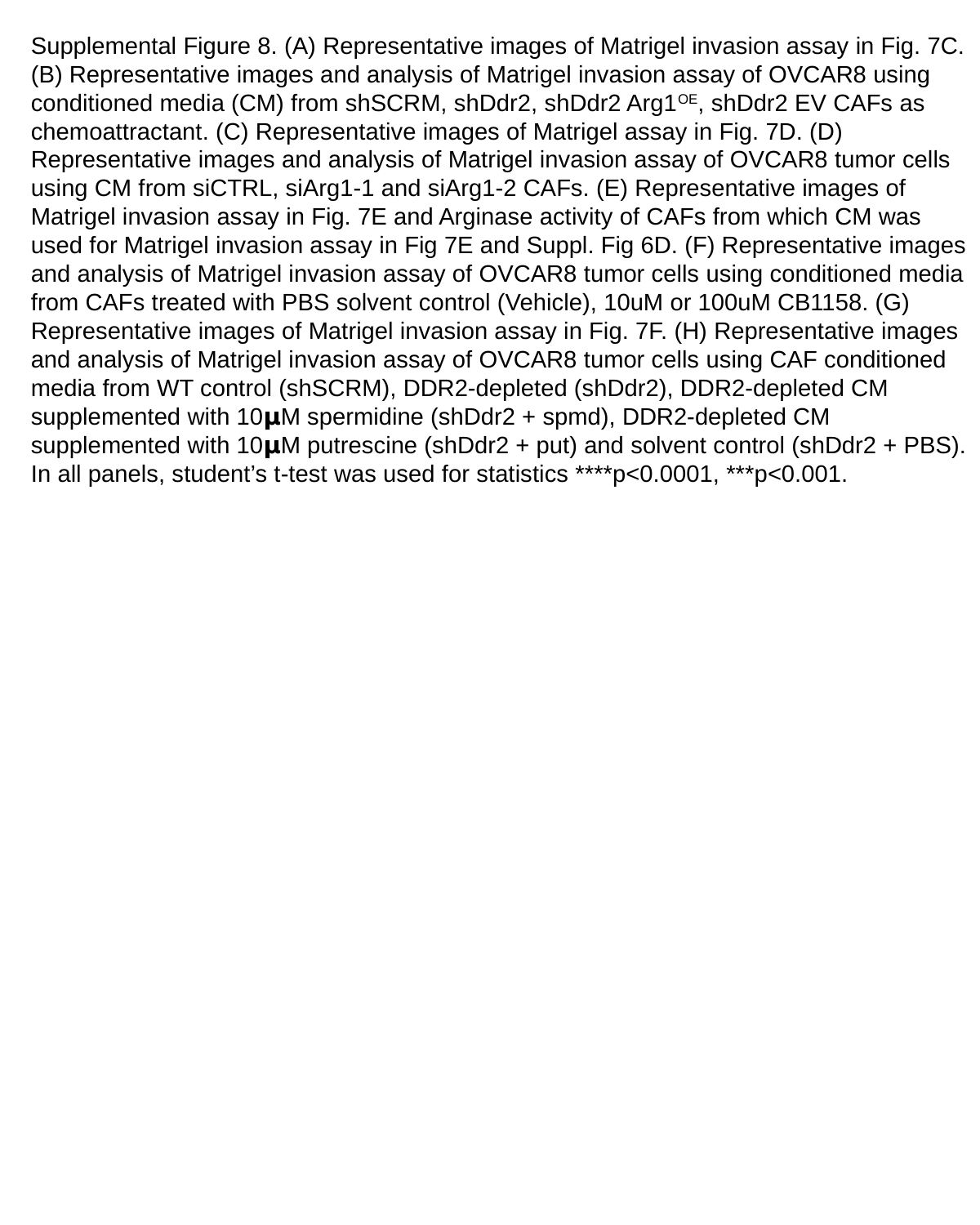

Supplemental Figure 8. (A) Representative images of Matrigel invasion assay in Fig. 7C. (B) Representative images and analysis of Matrigel invasion assay of OVCAR8 using conditioned media (CM) from shSCRM, shDdr2, shDdr2 Arg1OE, shDdr2 EV CAFs as chemoattractant. (C) Representative images of Matrigel assay in Fig. 7D. (D) Representative images and analysis of Matrigel invasion assay of OVCAR8 tumor cells using CM from siCTRL, siArg1-1 and siArg1-2 CAFs. (E) Representative images of Matrigel invasion assay in Fig. 7E and Arginase activity of CAFs from which CM was used for Matrigel invasion assay in Fig 7E and Suppl. Fig 6D. (F) Representative images and analysis of Matrigel invasion assay of OVCAR8 tumor cells using conditioned media from CAFs treated with PBS solvent control (Vehicle), 10uM or 100uM CB1158. (G) Representative images of Matrigel invasion assay in Fig. 7F. (H) Representative images and analysis of Matrigel invasion assay of OVCAR8 tumor cells using CAF conditioned media from WT control (shSCRM), DDR2-depleted (shDdr2), DDR2-depleted CM supplemented with 10𝝻M spermidine (shDdr2 + spmd), DDR2-depleted CM supplemented with 10𝝻M putrescine (shDdr2 + put) and solvent control (shDdr2 + PBS). In all panels, student’s t-test was used for statistics ****p<0.0001, ***p<0.001.

## Slide 12
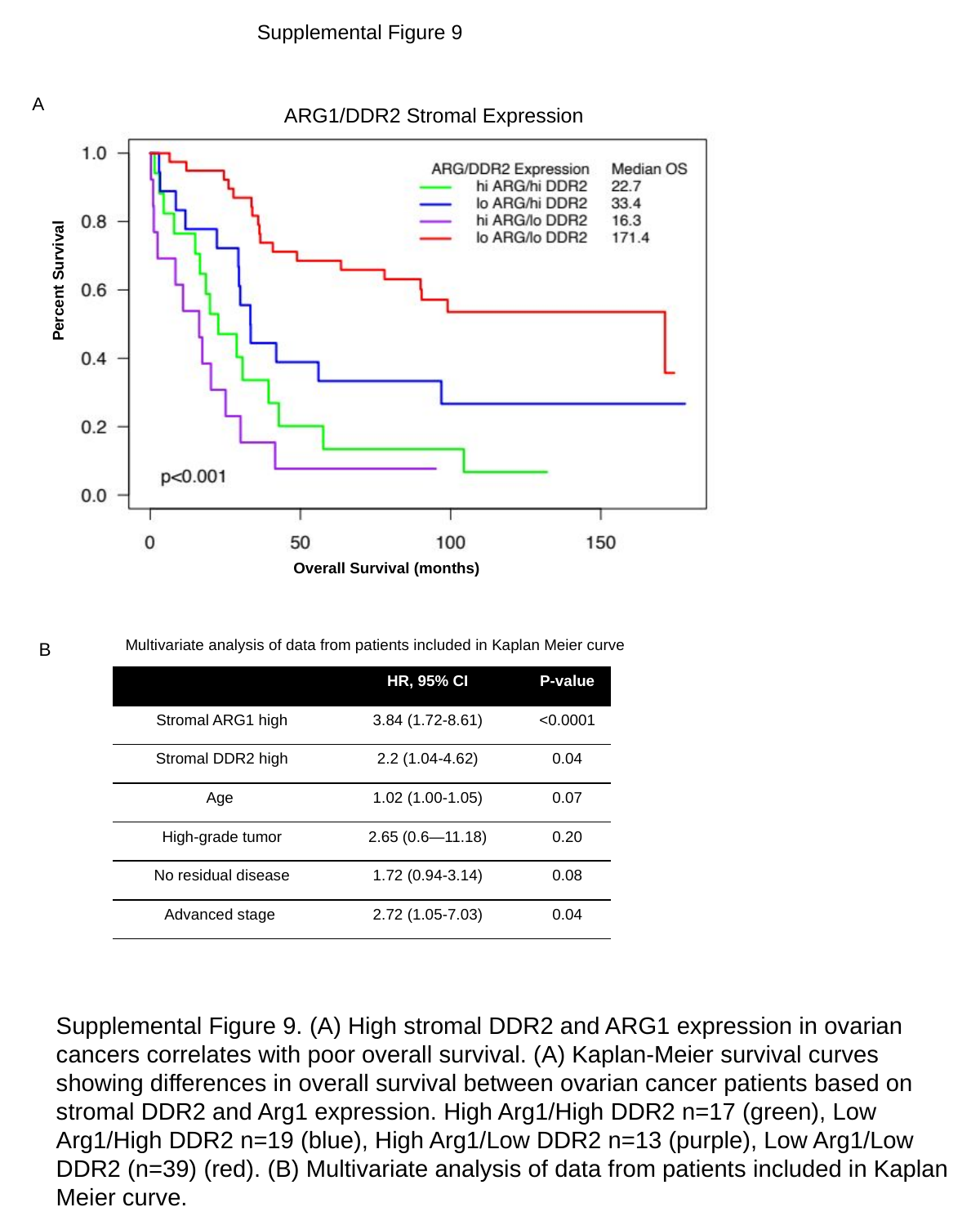

Supplemental Figure 9
A
ARG1/DDR2 Stromal Expression
Percent Survival
Overall Survival (months)
Multivariate analysis of data from patients included in Kaplan Meier curve
B
| | HR, 95% CI | P-value |
| --- | --- | --- |
| Stromal ARG1 high | 3.84 (1.72-8.61) | <0.0001 |
| Stromal DDR2 high | 2.2 (1.04-4.62) | 0.04 |
| Age | 1.02 (1.00-1.05) | 0.07 |
| High-grade tumor | 2.65 (0.6—11.18) | 0.20 |
| No residual disease | 1.72 (0.94-3.14) | 0.08 |
| Advanced stage | 2.72 (1.05-7.03) | 0.04 |
Supplemental Figure 9. (A) High stromal DDR2 and ARG1 expression in ovarian cancers correlates with poor overall survival. (A) Kaplan-Meier survival curves showing differences in overall survival between ovarian cancer patients based on stromal DDR2 and Arg1 expression. High Arg1/High DDR2 n=17 (green), Low Arg1/High DDR2 n=19 (blue), High Arg1/Low DDR2 n=13 (purple), Low Arg1/Low DDR2 (n=39) (red). (B) Multivariate analysis of data from patients included in Kaplan Meier curve.
