# Supplementary material for: DDR2-regulated arginase activity in ovarian cancer-associated fibroblasts promotes collagen production and tumor progression
Source: Oncogene. Author manuscript; Available in PMC 2024 Feb 16. (PMC10786713; doi:10.1038/s41388-023-02884-3)
Supplement: Supplementary Methods [file NIHMS1947626-supplement-Supplementary_Methods.docx]

Supplemental methods

Mouse experiments

For tumor burden experiments, 5 x 10^6^ ID8 *Trp53^-/-^ Brca2^-/-^* cells, 5 x 10^6^ KPCA cells or 2.5 x 10^6^ BPPNM cells were injected intraperitoneally into 8-12-week-old female C57BL/6J WT or Ddr2^-/-^ mice. Ddr2^+/-^ mice were bred to produce Ddr2^-/-^ mice and WT (Ddr2^+/+^) littermates (1). Genotype of mice was confirmed by performing PCR on mouse tail snips (Transnetyx primers: DDR2-2 WT and L1L2­Bact­P EX). Mice were sacrificed after 46 days for ID8 *Trp53^-/-^ Brca2^-/-^*, 30 days for BPPNM model and 19 days for KPCA model. Mice were monitored closely for signs of ascites or other symptoms. Mice were sacrificed via CO_2_ exposure and cervical dislocation. Dissection was performed and mesenteric and omental nodules were collected and weighed. For survival experiments, 5 x 10^6^ ID8 *Trp53^-/-^ Brca2^-/-^* cells were injected intraperitoneally into 8-12-week-old female C57BL/6J WT or Ddr2^-/-^ mice and mice were monitored until any predetermined endpoint was reached. Endpoint was defined using ascites accumulation, decreased activity, labored breathing and/or weight loss/gain >15% (2). For in-vivo colonization experiments, 1 x 10^6^ KPCA (luminescence positive) cells and/or 1 x 10^6^ mouse CAFs (luminescence negative) were injected intraperitoneally into 8-12-week-old female C57BL/6J WT mice. Arg1 constitutive over-expression was transfected into Ddr2^-/-^ mouse CAFs prior to co-injection. Mice were sacrificed 5 days after injection and omentum was collected for luciferase assay (Promega). Luminescence values were normalized to total protein concentration in sample as determined by Bradford assay.

NanoString nCounter mRNA expression profiling

The output for each sample was imported into nSolver Analysis System 4.0 (NanoString Technologies) using the Advanced Analysis package 2.0 and its custom analysis for quality control and analysis. Transcript counts were normalized using housekeeping genes. Genes with fold change >2 (log2 fold change >1) and p < 0.01 were considered significantly changed.

qPCR primers

RT-PCR primers used were as follows: *Arg1* mouse (Fwd 5’-ACA GCA AAG CAG ACA GAA CTA-3’, Rev 5’- GAA AGG AAC TGC TGG GAT ACA-3’), DDR2 human (Fwd 5′-TCA CCC AGA CCC ATG AAT AC-3′, Rev 5′-GGG AAG GAA ATG GCA TTA GG-3′), SNAIL human (Fwd 5'-TCG GAA GCC TAA CTA CAG CGA-3', Rev 5'-AGA TGA GCA TTG GCA GCG AG-3'), Arg1 human (Fwd 5’-CCC TTT GCT GAC ATC CCT AAT-3’, Rev 5’- GGC TGA TTC TTC CGT TCT TCT-3’).

Immunohistochemical staining and image analysis

Slides were mounted using Xylene-based Cytoseal (Thermo Fisher) or Vectamount (Vector Labs) as appropriate. For multiplex staining, slides were imaged, stripped with ethanol gradient and stored in 1X TBST until further use. Whole-tissue scans were obtained on a Zeiss Axio Scan Z1 Slide Scanner and analyzed with HALO software (Indica Labs) using Area quantification V1.0, Cytonuclear v1.5, or Cytonuclear FL v1.4 module.

Standard Activity and Metabolite Assays

40μl serum, 100mg crushed omental tumor or 1 x 10^6^ CAFs per well were used for arginase activity assay (Sigma MAK112). 1 x 10^6^ CAFs or 20 μl CAF conditioned media per well, diluted 1:200 after cell lysis and/or sample cleanup were used for L-arginine assay (Abcam ab241028). 1 x 10^6^ CAFs per well, diluted 1:100 after cell lysis and sample cleanup was used for Ornithine assay (Abcam ab252903). 1 x 10^6^ CAFs or 20 μl CAF conditioned media per well, 1:100 after cell lysis and/or sample cleanup were used for total polyamine assay (Sigma MAK349). 1 x 10^6^ CAFs per well was used for the hydroxyproline assay (Sigma MAK008). 100 μl CAF conditioned media per well (diluted 1:50 to remain in linear range) was used for the procollagen1a1 assay (R&D Systems DY6220-05). 1 x 10^6^ CAFs per replicate and 1ug total protein (normalized after homogenization) was used for proline assay (MAK427).

Single-cell RNA-sequencing and analysis

Samples were processed with each library sequenced on 0.125 NovaSeq S4 flow cell. Read alignment, gene expression estimation, normalization and quality control were performed using the Cell Ranger Single-Cell Software Suite (10x Genomics). Cell Ranger count was used to align samples to the reference genome (mm10), quantify reads and filter reads with a quality score below 30. Cell ranger output was imported into R and analyzed using Seurat package (3). For secondary analysis (4, 5) data from Gene Expression Omnibus was imported into R and analyzed using Seurat.

Genetic knockdown and overexpression

The oligos for human DDR2 shRNA, 5’- GCCAGATTTGTCCGGTTCATT-3’ and 5’-GCCAAGTGATTCTAGCATGTT-3’, and control, 5’- CCTAAGGTTAAGTCGCCCTCGCTC-3’, were cloned into the pLKO vector and infected cells were selected in puromycin (Sigma). The following siRNAs were used: siControl - ON-TARGETplus Non-targeting pool (Horizon Discovery D-001810-10-05), siARG1-1 - human ON-TARGETplus ARG1 siRNA SMARTpool (Horizon Discovery L-009922-00-0005), siARG1-2 - hs.Ri.ARG1.13.1 DsiRNA (IDT), siSNAIL-1 - human ON-TARGETplus SNAIL siRNA SMARTpool (Horizon Discovery L-010847-01-0005), siSNAIL-2 hs.Ri.SNAI1.13.1 DsiRNA (IDT). For ARG1 and SNAIL overexpression, DDR2 rescue and control vector, we used Arginase 1 (ARG1) (NM_000045) Human Tagged ORF Clone (Origene Technologies), Arginase 1/ARG1 cDNA ORF Clone, Mouse, C-DYKDDDDK (Flag®) tag (Sino Biological MG53754-CF), SNAIL/SNAI1 cDNA ORF Clone, Human, N-DYKDDDDK (Flag®) tag (Sino Biological HG16844-NF), DDR2 (NM_001014796) Human Tagged ORF Clone (Origene Technologies), and pCMV6-Entry (Empty) Mammalian Expression Vector (Origene Technologies) respectively.

Arginine tracing experiment

DDR2-expressing and DDR2-depleted CAFs were cultured in DMEM SILAC Flex Media (Gibco A2493901) supplemented with 10% dialyzed FBS (Gibco A3382001), 1% Penstrep, 25mM D-Glucose (Sigma G8270), 4mM L-Glutamine (Sigma G7513), 0.8mM L-Lysine (Sigma L5501) and 0.4mM 13C6 L-Arginine-HCL (Cambridge Isotope Laboratories CLM2265H-0.25). In parallel, CAFs were cultured in DMEM (Gibco 11965084) which contains unlabeled arginine and supplemented with 10% dialyzed FBS and 1% Penstrep. Cells were harvested at 72hrs and lysed in 0.5% SDS (BioRad 1610301), 50mM ammonium bicarbonate (Fisher A643500) and 50mM sodium chloride (Fisher BP3581) and 1X Halt protease inhibitor (Fisher PI78437) with ultrapure water (Fisher W6-4) and normalized for protein concentration.

The proteins were acetone/TCA precipitated and in-solution digested with trypsin. Digested peptides were desalted on C18 spin columns then subject to mass spec analysis. We searched the data against Homo sapiens database, using the MaxQuant search engine. Peptide quantification including 13C6-Arginine labeled peptide quantitation was performed based on the MS1 peptide intensity. The hydroxyproline, 13C5-hydroxyproline, and 13C5-proline were searched as modifications.

Polyamine detection by mass spectrometry (MS) - Analysis

Serum free conditioned media samples were diluted into 545µL of water and vortexed for 1 minute followed by heating at 60°C for 20 minutes. The internal standard was added to the sample, followed by sodium carbonate buffer (1M, pH 9) and isobutyl chloroformate to derivitize the polyamines (6). Samples were incubated at 35°C for 15 minutes, and the organic phase was extracted twice by adding 900µL of MTBE, samples were concentrated to remove organic solvent and then reconstituted in 45µL of MeOH. Polyamines were separated by reversed phase chromatography using an Agilent poroshell C18 column (2.7µm, 3.0 x 150mm). The column compartment was 35°C and the flow rate was set to at 0.100mL/min. All samples were kept at 4°C in the autosampler. Mobile phases were 100% acetonitrile (A) and water with 0.2% Acetic acid (B). The gradient was 50%B at 0 minutes, linear to 95% B at 12 minutes-17minutes then returning to 50% B by 18 minutes followed by a 10-minute wash between samples. Samples were analyzed an Agilent 6460 Triple quadrupole mass spectrometer coupled to an Agilent 1260 Infinity LC system. The following source parameters were used: gas temperature 250°C, capillary voltage 3,500V, drying gas flow 10L/min, sheath gas temperature 300°C, and nebulizer pressure 42 PSI. MRM parameters are shown in Supplemental table 8.

Matrigel invasion assay

Arginase inhibitor, CB1158, was purchased from ChemieTek and WT CAFs were treated overnight with 10 or 100µM CB1158 or solvent control (vehicle). CB1158 was removed from resultant conditioned media using a 10kDa molecular weight cutoff (MWCO) filter (Millipore UFC5010) before use as chemoattractant in invasion assay. For polyamine supplementation invasion assay, 10µM spermidine or putrescine or solvent control (vehicle) was added to DDR2-depleted CAF CM. CM from WT and DDR2-depleted CAFs was also collected and used in an invasion assay.

Chromatin Immunoprecipitation quantitative PCR (ChIP-qPCR)

For sonication step, optimized time of 15min was used. qPCR was used to determine gene expression levels after ChIP. qPCR primer design was done by finding putative Ebox regions upstream of Arg1 start site. ChIP-qPCR primers used for Arg1 were Fwd: 5’-GGGTGTGAAGTGAGAACATGA-3’ and Rev: 5’-TTCATACATGGAGTAACCTAACTCTG-3’. E-cadherin was used as a positive control for SNAI1 ChIP. Primers used for E-cad were Fwd: 5’-AGAGGGTCACCGCGTCTATG-3’ and Rev: 5’-AACTGCAAAGCACCTGTGAG-3’. Fold enrichment of gene expression was used for analysis using IgG ChIP samples as control.

Patient Survival Curve
At the time of tumor debulking surgery, samples were collected from patients with advanced stage, high-grade serous ovarian or fallopian tube cancer and used to create an ovarian cancer tissue microarray. Clinical characteristics and survival information were collected from patient charts. Overall survival was determined via Kaplan-Meier analysis using time of death or date of last patient follow-up.

The log-rank test was used for analysis and to differentiate the overall survival between patient groups. Using the ergodicity search (25%~75%), patients were sorted into two groups with low vs. high Arg1 and DDR2 expression and determined the log-rank P-values of overall survival and difference cutoff values. The value where the most significant P-value was determined to be the optimal cutoff level. Survival curves were calculated using the Kaplan–Meier method.

Collagen stimulation of CAFs

Collagen layer was formed overnight prior to culturing cells by placing a small layer (1ml) of pH-adjusted (7.0) collagen (1mg/ml) on 10cm plates and allowing polymerization at 37C overnight. DDR2-expressing and DDR2-depleted CAFs were cultured on plastic plates or 1mg/ml polymerized collagen layered plates for 24hrs. Cells were collected by scraping, lysed and western blot was performed for DDR2 expression.

References

1. Corsa CA, Brenot A, Grither WR, Van Hove S, Loza AJ, Zhang K, et al. The Action of Discoidin Domain Receptor 2 in Basal Tumor Cells and Stromal Cancer-Associated Fibroblasts Is Critical for Breast Cancer Metastasis. Cell Rep. 2016;15(11):2510-23.

2. Paster EV, Villines KA, Hickman DL. Endpoints for mouse abdominal tumor models: refinement of current criteria. Comp Med. 2009;59(3):234-41.

3. Hao Y, Hao S, Andersen-Nissen E, Mauck WM, III, Zheng S, Butler A, et al. Integrated analysis of multimodal single-cell data. Cell. 2021;184(13):3573-87.e29.

4. Iyer S, Zhang S, Yucel S, Horn H, Smith SG, Reinhardt F, et al. Genetically Defined Syngeneic Mouse Models of Ovarian Cancer as Tools for the Discovery of Combination Immunotherapy. Cancer Discovery. 2021;11(2):384-407.

5. Olbrecht S, Busschaert P, Qian J, Vanderstichele A, Loverix L, Van Gorp T, et al. High-grade serous tubo-ovarian cancer refined with single-cell RNA sequencing: specific cell subtypes influence survival and determine molecular subtype classification. Genome Med. 2021;13(1):111.

6. Magnes C, Fauland A, Gander E, Narath S, Ratzer M, Eisenberg T, et al. Polyamines in biological samples: Rapid and robust quantification by solid-phase extraction online-coupled to liquid chromatography–tandem mass spectrometry. Journal of Chromatography A. 2014;1331:44-51.
